# Mass-Balance-Consistent Geological Stock Accounting: A New Approach toward Sustainable Management of Mineral Resources

**DOI:** 10.1021/acs.est.3c03088

**Published:** 2024-01-02

**Authors:** Mark U. Simoni, Johannes A. Drielsma, Magnus Ericsson, Andrew G. Gunn, Sigurd Heiberg, Tom A. Heldal, Nedal T. Nassar, Evi Petavratzi, Daniel B. Müller

**Affiliations:** † Geological Survey of Norway, Leiv Eirikssons vei 39, 7040 Trondheim, Norway; ‡ Norwegian University of Science and Technology, Industrial Ecology Programme, Høgskoleringen 5, NO-7034 Trondheim, Norway; § Drielsma Resources Europe, 2585 GT The Hague, Netherlands; ∥ Luleå University of Technology, Department of Business Administration, Technology and Social Sciences, 97187 Luleå, Sweden; ⊥ British Geological Survey, Keyworth, Nottingham NG12 5GG, United Kingdom; # Petronavit AS, C/o Heiberg, Stokkahagen 23, 4022 Stavanger, Norway; ◆ U.S. Geological Survey, National Mineral Information Center, 12201 Sunrise Valley Dr., MS 988, Reston, Virginia 20192, United States

**Keywords:** mineral depletion, mineral resources, material flow analysis (MFA), sustainability reporting, United Nations Framework Classification for Resources (UNFC), digital economy, geoinformation management

## Abstract

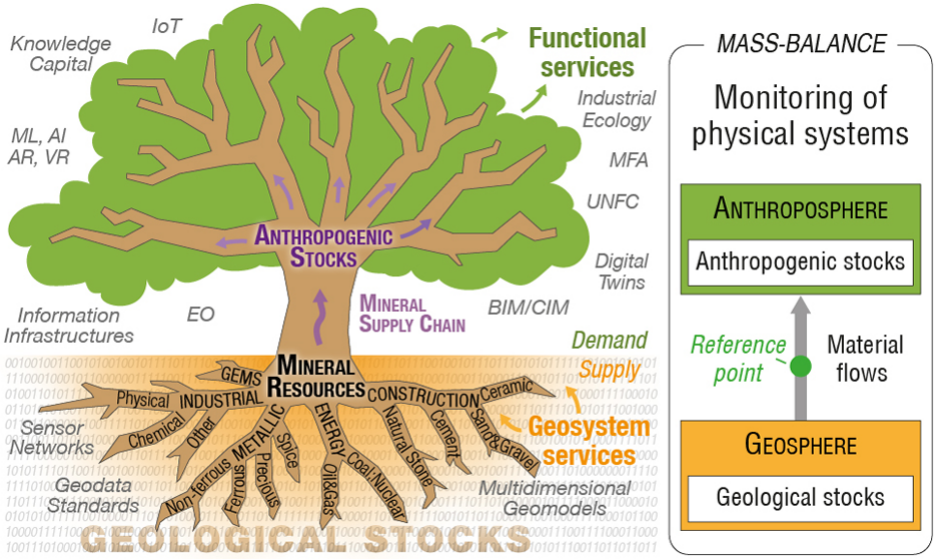

Global resource extraction raises concerns about environmental pressures and the security of mineral supply. Strategies to address these concerns depend on robust information on natural resource endowments, and on suitable methods to monitor and model their changes over time. However, current mineral resources and reserves reporting and accounting workflows are poorly suited for addressing mineral depletion or answering questions about the long-term sustainable supply. Our integrative review finds that the lack of a robust theoretical concept and framework for mass-balance (MB)-consistent geological stock accounting hinders systematic industry-government data integration, resource governance, and strategy development. We evaluate the existing literature on geological stock accounting, identify shortcomings of current monitoring of mine production, and outline a conceptual framework for MB-consistent system integration based on material flow analysis (MFA). Our synthesis shows that recent developments in Earth observation, geoinformation management, and sustainability reporting act as catalysts that make MB-consistent geological stock accounting increasingly feasible. We propose first steps for its implementation and anticipate that our perspective as “resource realists” will facilitate the integration of geological and anthropogenic material systems, help secure future mineral supply, and support the global sustainability transition.

## 1 Introduction

Are we running out of mineral resources? *Resource optimists* embrace the economic perspective of Smith,^1^ and others^2−5^ in arguing that markets are self-regulating, and that global mineral depletion is a nonissue^6,7^ for society. Indeed, while mineral extraction accelerated over the last century,^8^ there is little historical indication that physical depletion of individual deposits has impacted the global availability of minerals: inflation-corrected commodity prices remained stable over the past century,^4,9−11^ and commodity time-price trends indicate that mineral products became more affordable.^12^

*Resource pessimists* follow Malthus,^13^ Jevons,^14^ and Hubbert,^15^ in arguing that unconstrained mining will deplete Earth’s finite nonrenewable mineral stocks to the point of constraining future growth. The Club of Rome’s report “Limits to Growth”^16^ intensified the discussion about finite stocks.^17−19^ While its predictions of physical scarcity have not materialized,^20^ the depletion of deposits indeed accelerated together with extraction rates,^21,22^ ore grades in production declined,^23,24^ and environmental impacts and resource conflicts multiplied and intensified.^22,25−29^ Global trends continue to raise broad concerns about future raw material availability and sustainability.^21,30−33^

The mineral depletion and sustainability debate continues unabated, and while neither the optimistic nor the pessimistic perspective is inherently contradictory or incorrect, neither has offered a unifying solution to reconcile the positions.^4,34−39^ Notably, both resource optimists and resource pessimists base their claims on the same national and global mineral production statistics and estimates for mineral resources and reserves.^40−42^ Whether these data are at all suitable for quantifying long-term mineral depletion is questioned by recent studies.^11,43−46^ Various authors highlight significant uncertainties regarding conceptual methods for estimation, classification, and spatial aggregation of mineral resources and reserves across all data sources, particularly for critical raw materials.^47−51^ Moreover, the general lack of systematic and standardized granular mine-site-level “bottom-up” information is a key concern for comparing, aggregating, and monitoring mineral resources and mineral reserves.^42,51,52^ Poor data availability also hampers environmental, social, and governance (ESG) risk assessments,^53,54^ sustainability analysis,^55,56^ and raw materials scenario modeling.^47,53,54^ Notwithstanding, there are few recommendations on how mineral-related data collection may be streamlined and industry-government integration facilitated to address data gaps and fragmentation. Here, we use material flow analysis (MFA) and mass-balance (MB) principles to review current mineral reserve accounting, mine production monitoring, and industry-government data integration. We use this background to define a MB-consistent geological stock accounting approach and broader framework that can help to establish a coherent language across the relevant research fields. We will illustrate how our approach may be used to address the identified data gaps and data fragmentation, and review its context within recent trends in Earth Systems modeling and policymaking to propose next steps toward physical accounting and material systems integration.

## 2 A Brief Review of Mass-Balance-Consistent Accounting

Monitoring dynamic changes of physical stocks and flows of materials and energy in the *socioeconomic metabolism*(57) or *physical economy*(58) reveals how we as producers and consumers of goods and services depend on, and shape, the anthropogenic and natural environment. The birth of *industrial dynamics*(59) in the late 1950s, *industrial metabolism*(60,61) in the 1980s, and *industrial ecology*(62,63) in the 1990s, have laid the groundwork for using integrated system approaches as tools for natural resource management, circular economy efforts, and sustainable development.^64−66^ Since the first formal “materials balance approach” of the U.S. economy was published in 1969,^67^ MFA has become a well-established method for modeling anthropogenic and natural physical systems at multiple scales, from single-unit processes at the facility level, to complex global material and energy systems.^68,69^ MFA builds on the basic principle of conservation of mass, derived from the First Law of Thermodynamics.^70,71^ Throughout history, conservation of mass (MB-consistency) has been recognized as fundamental in chemistry,^72,73^ forestry,^74−76^ glaciology,^77−79^ hydrology,^80−82^ climatology,^83,84^ as well as in geology,^85−87^ petroleum reservoir modeling,^88,89^ mineral processing,^90,91^ and urban metabolism studies.^92,93^ MFA formalizes MB-consistency for physical accounting (materials accounting) by requiring that (i) the system boundary be explicitly defined in space and time, (ii) stocks and flows be expressed in consistent physical (nonmonetary) units, and (iii) mass and energy be in balance across transformation, distribution, and storage processes in the system.^68,69^ Materials occupy space and can only be accounted for if the system boundary and the processes are clearly defined in space (3D) and time,^70^ e.g., to quantify natural groundwater flows in the Earth’s subsurface, or to model the material stock in houses in the built environment. 2D geospatial data are insufficient, as they can only indicate where materials are on a map but cannot capture their physical characteristics (e.g., 3D shape and extent, mineral distribution, overburden thickness) or their material balance volumes.

For analyzing physical systems, a MB-consistent MFA approach brings diverse benefits:^70,71,94^

- The system structure of connected flows carries additional information about the origin and destination of the flows.
- Mass balance equations make the system structure explicit and can close data gaps without requiring additional data collection.
- The explicit system definition allows for balancing each process for total mass and all chemical elements, and facilitates data harmonization and integration (e.g., to avoid double-counting).
- The MB principle is useful for sensitivity analysis and data reconciliation. It enables robust accounting and scenario models for physical matter in the “real world”.

**Figure 1:**
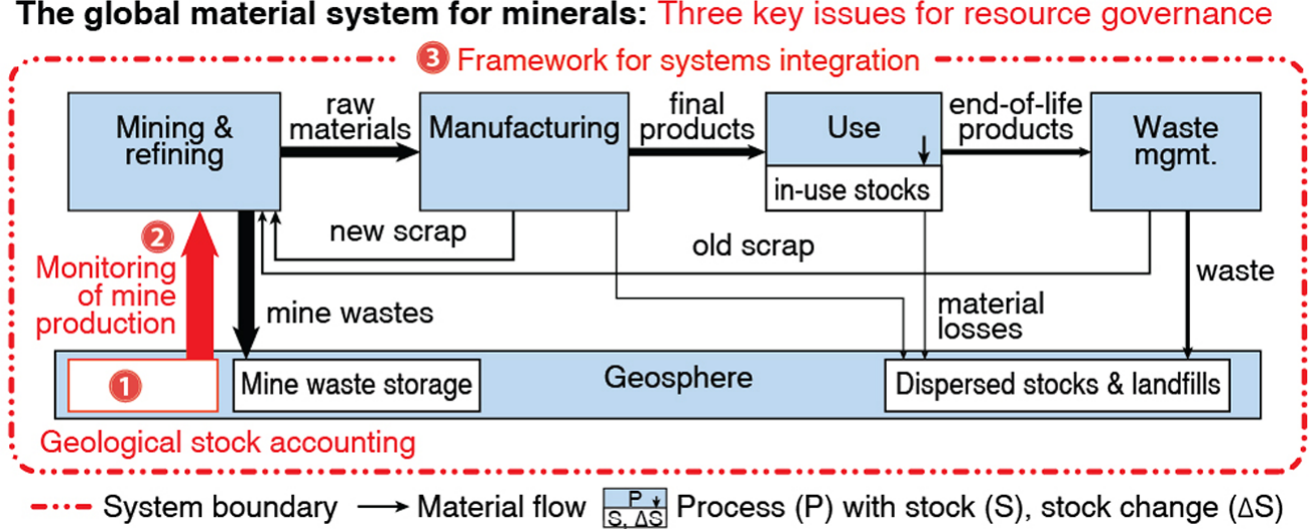
Simplified material flow analysis (MFA) system of the global mineral material cycle. *Material flows (arrows)* connect material transformation, transport, market, and storage *processes (blue boxes)* with or without *material stocks (white boxes)*. Highlights in red identify three key issues that require mass-balance-consistent mineral information: geological stock accounting (section 3), monitoring of mine production (section 4), and physical systems integration (sections 5 and 6).

Altogether, MFA provides a robust and transparent reference framework to understand, visualize, and transform material and energy systems and their associated value, information, and emission layers toward sustainability.^94−96^ Recent applications include a plant-level study for Europe’s biggest aluminum smelter outside of Russia,^97^ the International Aluminium Institute’s “Global Aluminium Cycle”,^98^ the European Union’s “Material System Analysis” studies,^99^ and U.S. Geological Survey publications on tantalum, niobium, and rare earth elements (REEs).^100−102^ While there are many examples to demonstrate MFA’s utility, there has not been much discussion about MFA in mineral resource geology, mineral depletion studies, and sustainable mining (cf. Supporting Information, S1.3), partly because of data gaps and a lack of transparency in current industry minerals reporting. Data fragmentation and poor international harmonization^8,41,42,51^ impede MB-consistent physical accounting. Here, we address this gap and use MFA principles to discuss three key issues related to (1) geological stock accounting (section 3), (2) monitoring of mine production flows (section 4), and (3) systems integration (sections 5 and 6), as illustrated in Figure 1.

## 3 The Concept of Geological Stocks

**Figure 2:**
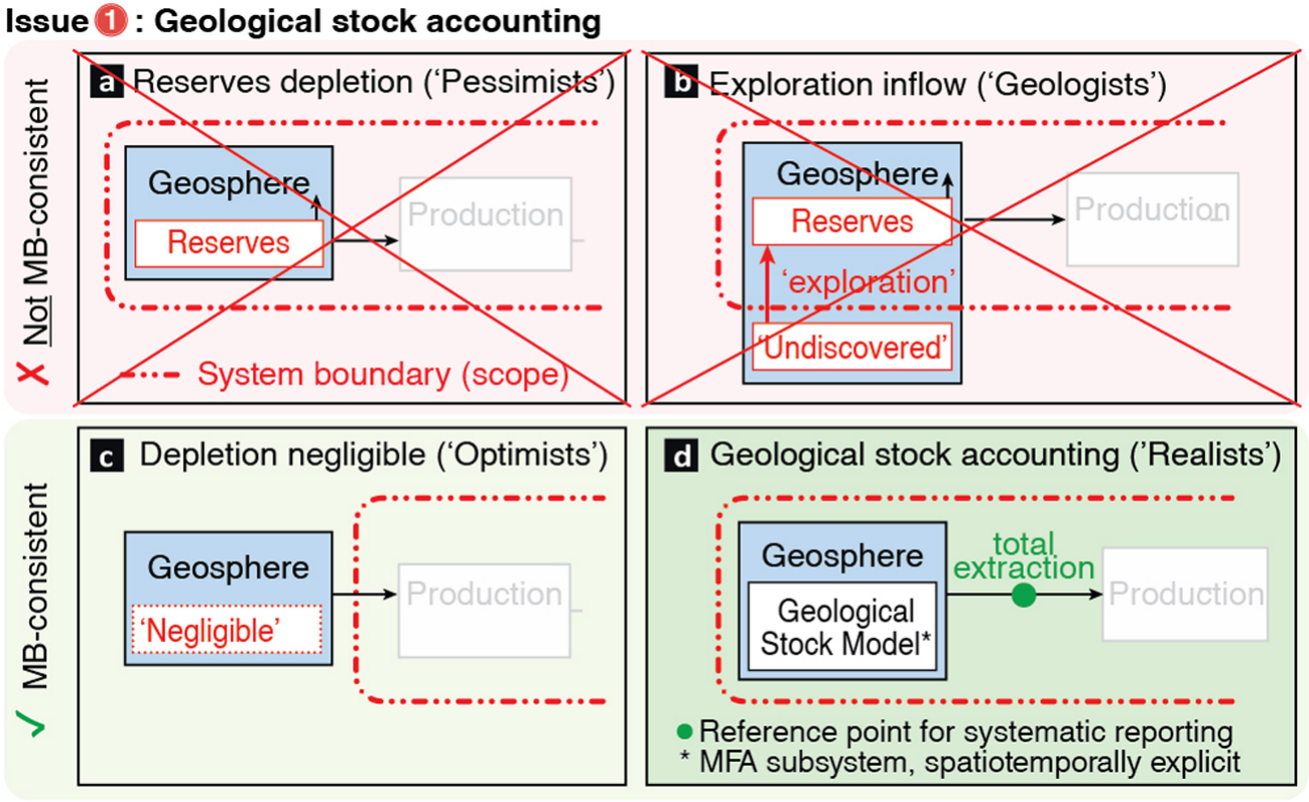
Different approaches for geological stock accounting: (a) reserves included as fixed stocks within the system boundary; (b) exploration interpreted as a (in)flow of material; (c) geosphere excluded from the system boundary; (d) multidimensional and mass-balance (MB)-consistent geological stock model. Approaches (a) and (b) violate material flow analysis (MFA) principles, (c) is permissible but uninformative, and (d) is the spatiotemporally explicit conceptual approach.

Mineral deposits are nonrenewable in human time scales, and geological exploration and mine project development need to compensate for deposits being depleted. This is increasingly challenging, as global demand for minerals continues to grow,^22^ while the probability of exploration projects reaching the mining stage remains notoriously low.^20^ Moreover, lead times from prospecting to production often reach 8–11 years for exploration and additional 9–12 years for mine development.^103^ Government-industry information flows are crucial in this context. Definitions, methods, and standards for government and industry data collection, sharing, and integration, however, have diverse historical backgrounds and have been developed by different organizations to serve distinct information needs: Government agencies, for instance, collect basic geoscientific and industry data to inform long-term resource management, promote sustainable development, and secure an affordable supply of raw materials at national, regional, and local levels. *National- and regional-scale* geospatial data sets and mineral resource estimates by government agencies often cover both known and undiscovered mineral deposits, and aim to inform a wide range of users including policy makers, exploration companies, and investors.^42,104−107^ These data sets and resource estimates, however, have diverse underlying assumptions, varying uncertainty, and significant data gaps,^41,50,108,109^ which make them difficult to compare and integrate. Exploration and mining companies, on the other hand, collect detailed *site-scale* information for project-specific appraisal (valuation) and operations planning, with the goal of generating revenue though successful mining and refining ventures (Figure 1). As early stage industry exploration projects advance to drilling, permitting, and construction, their costs rapidly grow,^20^ and companies may use public disclosure to report promising exploration results and attract capital for project development. The 1997 Bre-X mining fraud, which cost investors US$6 billion,^110^ led regulators and professional associations to increasingly require that such industry disclosures follow “resource classification standards”^108,111^ (cf. S2) overseen by a certified professional, known as competent person (CP)^112^ or qualified expert (QE).^108,111,113^ While the definitions of the terms *resources* and *reserves* vary across extractive industries (e.g., industrial minerals, metals, oil and gas) and between jurisdictions (e.g., Australia, Canada, China, United States),^108,111,114^ they are commonly understood along following lines:^104,115^*reserves* are the amount of discovered in-ground commodities that are considered to be *economically recoverable and marketable at the time of determination through projects that are committed to be realized*, while *resources* comprise both discovered and undiscovered quantities that *are not yet economically recoverable at present in this sense, but may, eventually, be extracted*. Both definitions presume some degree of recoverability and human intent, which indicates that resources and reserves are a function of exploration efforts, market demand, regulations, and other environmental, socioeconomic and technical variables (collectively often called “modifying factors”^50,112^). Reserves are by definition better constrained than resources, yet both are somewhat uncertain, and inherently dynamic.^108,109^ Remembering this commonality, we here simply use “reserves” to refer to reported quantities estimated through national and international (e.g., CRIRSCO-aligned)^112^ resource classification standards or through the more generic United Nations Framework Classification for Resources UNFC.^116^ In contrast to project- and commodity-specific classification of reserves (i.e., reporting only the amount of commodity x we can mine and sell for profit or by applying subsidies), the scope for MB-consistent geological stock accounting is broader in that it aims to facilitate *spatiotemporally explicit* and *MB-consistent* data integration and physical accounting for the *entire* geological “subsurface”^117^ of a geographical region. Using the terms *stock*,^70,118^*intrinsic physical properties*,^119,120^*spatial compartment*,^68,70^ and *mutually exclusive and collectively exhaustive (MECE)*,^119^ we suggest the following new definitions: *the geological stock is the physical content of a natural material compartment that is delimited by a spatiotemporally explicit, georeferenced, and time-invariant 3D system boundary. Geological stock quantification* maps the material content in the defined compartment in a *mutually exclusive and collectively exhaustive* manner at a specified *reference point* in time, based on purely *intrinsic (e.g., physical, chemical, and mechanical) material properties*. Intrinsic properties include, for instance, the total mass, elemental composition, mineralogy, and strength. A physical *material flow* out of the defined compartment occurs when natural material leaves the system boundary during the period of consideration between two reference points in time, independent of whether the material has an economic value, and regardless of whether the flow results from human activity or natural processes. *Geological stock accounting* tracks physical material stocks and flows and their changes over time based on their purely physical attributes, which makes it conceptually suitable for MB-consistent raw material system modeling and scenario development. To illustrate conceptual differences between mineral resource classification and MB-consistent physical accounting, we use four MFA system definitions (Figure 2), and individually discuss them below.

### 3.1 Using Reserves to Calculate Depletion (Figure 2a)

National and global resources and reserves numbers are often combined with production statistics to calculate mineral depletion rates.^16,35,36,38,39,121^ This incorrectly assumes, without explicitly stating so, that reserves are fixed physical quantities, i.e., “*all there is”*.^111^ Accelerating global resource extraction would thus progressively deplete them, aligning with the “fixed-stock paradigm”^4^ and pessimistic predictions of impending global exhaustion. However, Zimmermann^122^ pointedly stated in 1951: “resources are not, they become; they are not static, but expand and contract in response to human wants and human actions”. Indeed, reported global resources and reserves of many commodities continue to grow, despite accelerating extraction rates.^107,123,124^ That reserves are inherently dynamic^125^ is illustrated by two examples: first, mine life cycle disruptions such as bankruptcy can unexpectedly and instantaneously “erase” a company’s reserves, reducing national (and global) reserve totals; and second, more efficient technology can turn previously subeconomic parts of a deposit into economic reserves, thus increasing reserve totals. Notably, long-term commodity prices have remained relatively stable^4,9,11^ despite declining ore grades in production.^23,24^ This was predominantly driven by exploration successes, technological innovation, and economies of scale, which lowered the threshold for economic mining and increased global reserves.^23,126,127^ On a project level, reserve calculations commonly use a cutoff grade to estimate recoverable in-ground quantities, defined by a set of assumed operating conditions with variable uncertainty, including technical feasibility, labor and fuel costs, taxes, and projected commodity prices. Changes to any of these may call for adjusting the cutoff grade. Higher market prices, for instance, make it feasible to profitably mine lower-grade and deeper ores. Interestingly, empirical data suggest that there is no specific geological or thermodynamic grade-threshold that may limit this trend. While geologists previously postulated a “mineralogical barrier” for the copper concentration (copper ore grade) in the crust,^128,129^ randomly sampled data across all rock types indicate a unimodal continuum.^130^ The absence of a clearly identifiable *intrinsic*(119) ore grade threshold emphasizes that the definition of “ore deposits” (i.e., naturally occurring mineral material “known to be producible to yield a profit*”*)^131^ is arbitrary from a physical accounting perspective. Reserve numbers of individual industry projects can thus be understood as a snapshot of a “working inventory”^132^ that dynamically evolves in function of socioeconomic (and thus *extrinsic*(119)) factors. In addition, project owners may choose or be required to selectively disclose only some of their reserves, to the extent that fits their commercial interest and applicable regulations. Arguably, project owners theoretically have the information to “account” for the entire 3D geological stock volume in their concession area in a MB-consistent manner (e.g., by using 3D block models and data reconciliation).^133,134^ However, their published reserve numbers only represent those selected individual 3D “blocks”^20,135^ that fulfill the reserve classification criteria (i.e., a dynamically evolving subsample of the total geological stock). All 3D information is lost after reporting, which implies that published reserves become decoupled in space and time and do not allow for MB-consistent data integration, reconciliation, and material stock and flow accounting. Government mineral inventories and national reserve totals compiled from these selectively reported industry reserve quantities (plus possibly additional government estimates) are hence poorly suited for MB-consistent physical stock accounting. Altogether, while previous authors have already pointed out that reported reserve numbers should not be misinterpreted as fixed stocks,^7,11,111,122^ we here show that doing so violates MFA principles, and that these estimates cannot be used for MB-consistent physical accounting because reserves data (i) lack explicit georeferencing and a time-invariant 3D system boundary (cf. spatial compartment); (ii) are inherently dynamic and co-defined by extrinsic socioeconomic factors (which continuously changes the MFA balance volume); (iii) and are selectively sampled and neither mutually exclusive nor collectively exhaustive across time and space.

### 3.2 Modeling Exploration as an Inflow (Figure 2b)

To account for the dynamic nature of reserves, it has been suggested to introduce exploration as an imaginary *inflow*(136) (“exploration” arrow) representing *“flows from unknown resources into a reserve inventory”*.^7^ Geologists commonly categorize exploration projects into greenfields and brownfields exploration, and aim to provide information that helps to “convert”^137^ or “upgrade”^125^ mineral discoveries into mineable reserves. Conceptually, greenfield exploration *expands the system boundary* of reserves though new discoveries and classification *outside* of previously known geological districts or terrains. This changes the balance volume during the accounting period, which makes it impossible to uphold the MB-principle because the 3D system boundary (spatial compartment) is not time-invariant.^70^ Brownfields exploration, in contrast, *increases the knowledge within* previously known terrains that have existing data, often in the vicinity of abandoned or operating mines. New measurements and subsequent (re)classification^20^ update the geological knowledge of individual blocks inside the imagined 3D system boundary around the entire brownfields volume, and may increase or decrease reported reserves. Regardless, exploration is no measurable physical flow, and this approach cannot solve the MB-consistency issues inherent to reserves accounting.

### 3.3 MB-Consistency without Geological Stocks (Figure 2c)

The resource optimist’s view can be framed as geological stocks being so vast and markets and human ingenuity so successful in developing new solutions, that accurate quantification of geological stocks is simply irrelevant.^2−5,138^ In other words: “Whatever is left in the ground is unknown, probably unknowable, but surely unimportant; a geological fact of no economic interest”.^7^ Indeed, geologists point out that the mineral content of the Earth’s crust is orders of magnitude bigger than reported industry reserves.^111,130^ Economists may argue that functioning markets automatically balance production and consumption, and that focusing on production costs and prices, and addressing market failures, is more important than quantifying physical availability.^4,139^ Translated to MFA, this approach draws the system boundary such that the geological subsystem (geosphere) is excluded from consideration. While this is indeed a MB-consistent system definition, it does not contribute to tracking how Earth’s natural resources are depleted. Importantly, it does not contribute to data collection and knowledge integration for “physically consistent”^140^ modeling and Earth System Science,^141^ which we need to evaluate prospective mining localities, develop supply scenarios, and address the ESG issues that are likely to limit mineral production well before any global physical depletion.^132,142,143^

### 3.4 MB-Consistent Geological Stock Accounting (Figure 2d)

MB-consistent geological stock accounting requires a spatiotemporally explicit system definition to describe and monitor changes in the geological stock volume in the geosphere. Here, we propose to model the geological stock using a full-coverage 3D digital geomodel (cf. section 5) with a georeferenced time-invariant (fixed) spatial system boundary that establishes the model’s initial physical *reference state*(27) at an initial *reference point* in time. By recording the intrinsic physical material properties of stocks and separating them from socioeconomic (and thus extrinsic)^119^ factors, we can use MFA to track the actual physical changes over time. Combining spatial resolution and MB-equations facilitates both site-specific and regional-scale geological stock quantification and MFA data integration (MFA subsystem modeling approach).^144^Section 5 elaborates how changes in geological stocks due to mining and better knowledge about intrinsic material properties of specific blocks can be represented. Moreover, this method considers the entire 3D distribution of geological materials in the Earth’s crust and its uncertainty, not just the continuously changing reserves in known mineral deposits. Notably, this scope definition also satisfies the stated objective of the UN System of Environmental-Economic Accounting (SEEA)^145^ “to include all of the resources that may provide benefits to humanity” (i.e., the entire 3D geological stock), while it also “allows for a full analysis of changes”.^145^ Altogether, our geological stock accounting approach aims to facilitate data integration to represent the physical reality as accurately as possible with a continuously increasing resolution.

## 4 Physical Monitoring of Mine Production

**Figure 3:**
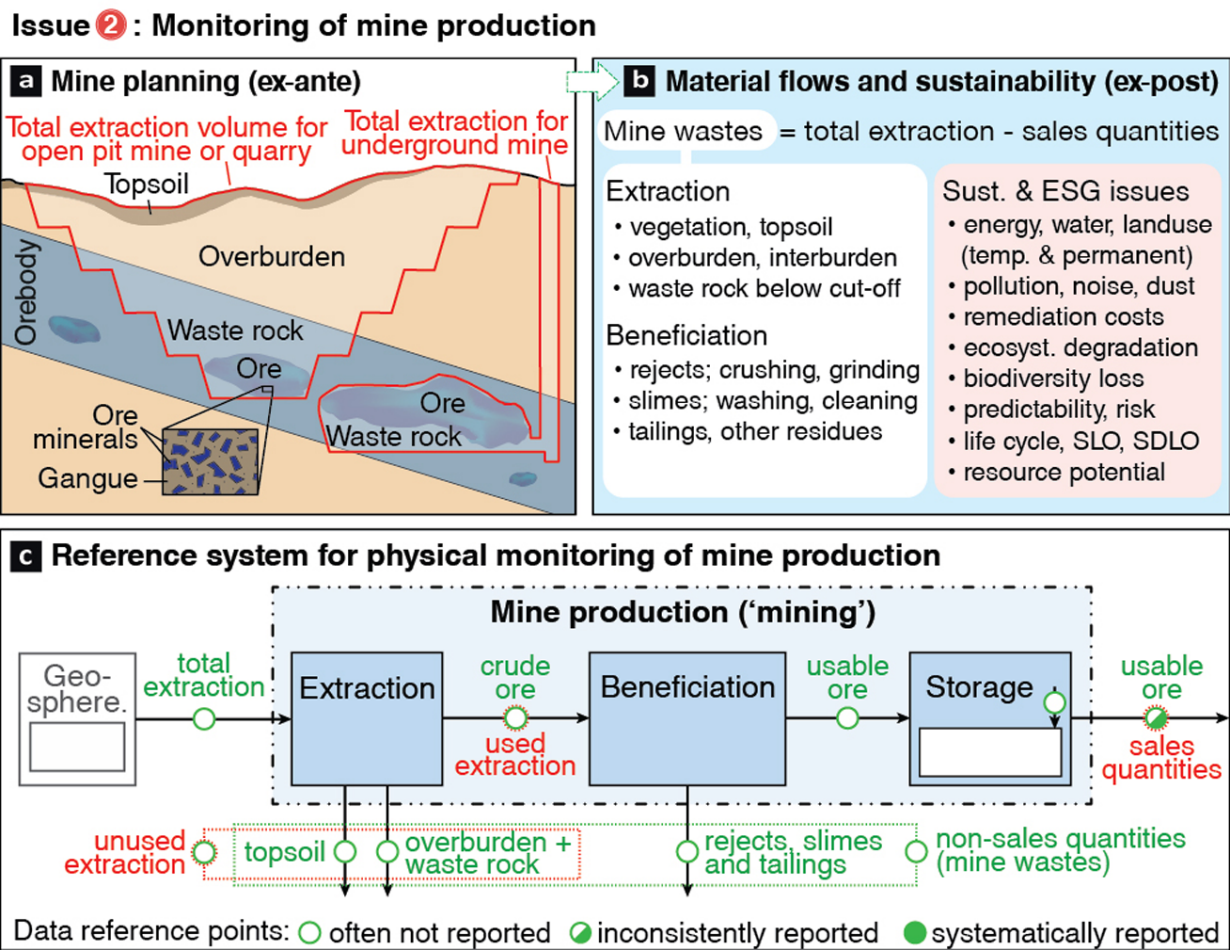
Physical monitoring of mine production. (a) Mine planning: The natural characteristics of mineral deposits such as depth and ore grade, combined with mine design and operating efficiency, determine the expected (ex-ante) material flows. Figure not to scale, modified after ref (163). (b) Material flows and sustainability: Material flows of mining are interlinked with environmental, social, and governance (ESG) issues and tracking them is thus crucial for the Social License to Operate (SLO) and Sustainable Development License to Operate (SDLO). (c) Reference system for physical monitoring: A standardized material flow analysis (MFA) system definition with explicit reference points and a mutually agreed-upon terminology facilitates systematic reporting and enables mass-balance-consistent monitoring of mine production flows.

It is widely accepted that granular disclosure of relevant data is a key driver for responsible mining and achieving the Sustainable Development Goals (SDGs), for example decent work and economic growth (SDG 8), and responsible consumption and production (SDG 12).^146,147^ However, systematic site-scale information is still “conspicuously missing”^148^ from corporate sustainability reporting of mining companies, while corresponding government data sets are often incomplete, fragmented across different agencies, and difficult to integrate, as we show in Figure 3 and the following sections.

### 4.1 Industry Reporting of Mine Production Flows

Mining companies routinely collect material flow data at different stages of their mine life cycles to manage their economic efficiency^149^ and ESG risks.^150,151^ However, public disclosure of data on observed past (ex-post) or expected future (ex-ante) material flows is rare and typically neither systematic nor MB-consistent. The amount and quality of in-house information increases as projects advance along the project-production cycle:^152^ during the prefeasibility and feasibility stages, expected sales production and waste flows can be estimated by combining geological information with mine design and scheduling.^153^ This makes it necessary to characterize in-ground materials and to calculate total extraction (excavation) volumes and associated flows of topsoil, overburden, below cutoff grade waste rock, and pay-grade ore for further processing (Figure 3a). Besides being essential for mine life cycle costing and environmental optimization at a corporate management level,^154,155^ this ex-ante information is also of interest for national mineral resource governance and local stakeholders: Specifically, it could be used to demonstrate compliance with legal, regulatory, contractual, fiscal, and infrastructural requirements, and could facilitate stakeholder negotiations to define the terms of the “Social License to Operate” (SLO) and “Sustainable Development License to Operate” (SDLO).^53,148,156^ Ex-ante data could also help exploit synergies^71^ between different projects at an early planning stage where design decisions can still be influenced. Yet, these data are rarely systematically reported or updated to reflect changes in planning. During the actual production phase(s), companies routinely monitor material flows along the processing chain to manage operations and ESG risks (Figure 3b).^91,133,134^ Many jurisdictions mandate annual reporting of “mine production” or “sold production” quantities to mining authorities. However, reported data (information flows) are typically not defined with MB-consistent *reference systems* (e.g., Figure 3c). This results in misunderstandings regarding what reported production data refer to (e.g., the total mass with average ore grade, or the total pure metal content of sold products) and allows for “hidden” inconsistencies. Moreover, published industry data generally only cover some selected materials and flows (e.g., omit to report removed waste rock),^40^ exclude relevant details (e.g., whole-rock composition including companion or critical metals and deleterious elements; mineralogy; pH; physical product qualities),^50,157^ and may be preaggregated across projects to company-levels (i.e., not granular, site-scale). Moreover, depending on the jurisdiction, they may remain entirely undisclosed for entities that are not listed on stock exchanges, or that have revenues below a given threshold.^158,159^ Similarly, government communication is a problem.^160^ Reporting may for instance be fragmented across agencies: mining directorates, tax authorities, national environmental protection agencies, or local municipal planning offices collect (but not necessarily share) ex-ante or ex-post information on mining-related material flows for environmental impact assessments, license extensions, taxation, and closure procedures. Similarly, information on planned or completed excavation for urban and infrastructure development projects are often poorly integrated with information on mining projects,^161^ despite being relevant for construction aggregates (“development minerals”) management and circular economy strategies.^162^ Altogether, current production reporting does not provide complete material flow data coverage and lacks a material systems context.

### 4.2 Government Aggregation of Production Data

Reported mine production data are commonly ingested by geological survey organizations (GSOs), mining directorates, and industry associations. Due to confidentiality concerns, these organizations usually only publish them as aggregated mineral production statistics.^40−42,158^ National mine production totals and global production estimates are used by a wide range of stakeholders, e.g., to evaluate markets for project development, assess raw material criticality,^164−168^ investigate the long-run availability of metals,^43,105^ and develop raw material policies and science-based resource efficiency targets.^169^ However, the published production statistics are often misinterpreted by data users that do not know their context. Back-calculations using published production statistics, for instance, systematically underestimate total extraction^46^ because the quantities, types, and composition of nonsales material flows are not correctly reported. Historically, the flows of topsoil, over- and interburden, and below-cutoff waste rock, collectively referred to as *hidden* or *indirect* flows,^170^*unused extraction*,^171^ or *natural resource residuals*,^145^ were not considered as tradeable commodities with economic value^46,67,172,173^ and are thus not reported. Indeed, national accounting systems including Eurostat (cf. “Waste disposal to the environment”)^171^ and the UN System of Environmental-Economic Accounting (SEEA)^145^ still consider them to be “outside” of the system boundary, assuming they are immediately returned to and part of “the environment”. This scope partly explains why quantitative data on unused extraction are absent from national statistics.^46,174^ Yet, this does not lessen their relevance for sustainability-related discussions. Hidden flows exert various pressures on the environment and can contain both potentially harmful and useful material.^175^ For open pit mines, hidden flows are commonly two, and occasionally 30 times bigger than the ore retained (used extraction),^176^ and orders of magnitude bigger than final sales quantities.^40,177^ Globally, the mining industry is the largest “waste” producer,^156^ and in 2016 alone the flow of unreported waste rock was estimated to be 72 billion tonnes (Gt).^178^ Altogether, the historical flow of *nonsales* quantities (hidden flows, reported tailings, and other residues) is estimated to have accumulated a total of several hundred Gt of mine wastes.^179^ While nonsales quantities raise various ESG issues, they can also offer opportunities for remining of tailings, ecosystem restoration, and higher-value land use (Figure 3b). All countries with important mining histories have legacy mine waste stocks. In the United States alone, there are estimated 550 000 abandoned mines, 4–13% of which may pose a risk to human health and the environment.^180−182^ The estimated remediation costs of the 64 priority sites are US$7.8 billion, of which $2.4 billion would come from taxpayers.^183^ Notably, many risk-prone historical practices have been superseded,^184^ and historical extraction rates used to be much lower than today. The accelerating mineral extraction rates^21,22^ and increasing waste-to-ore ratios,^51^ on the other hand, spotlight the need to better understand and address future waste flows, including though transparent reporting of tailings storage^178,185^ and monitoring of unused extraction (e.g., topsoil, over- and interburden, waste rock; Figure 3b). This emphasizes concepts such as *circular economy*,^186^*zero waste*,^187^*comprehensive extraction/comprehensive resource recovery*,^188,189^ in addition to *remining, reprocessing,* and *rehabilitation*.^184,190^ All require *site-scale* and MB-consistent data on material flows and stocks to identify ESG risks,^191^ facilitate sustainable sourcing, evaluate residual resources and market potentials,^192^ and to allow for robust data integration. Still, data gaps on mine waste types, volumes, mineralogy, and composition continue to impede resource recovery from growing waste streams (“*mining*” *of flows)* and historical waste deposits^193^ (*remining of legacy stocks*). Similarly, Economy-Wide Material Flow Accounts^194^ and indicators for Material Footprints,^195^ Total Material Requirements,^172,173^ Rock-to-Metal Ratios,^40^ and project-specific resource efficiency^54,196^ are all hindered by the poor availability or lack of relevant, robust, and accessible site-scale material stock and flow data.

### 4.3 Reference Systems for Consistent Reporting

MB-consistent material systems are useful for defining terms (e.g., stocks, flows), relationships, and indicators (e.g., circularity, efficiency, recovery rate). Mining has a long and diverse history across the world, and conflicting definitions abound. The term *ore*, for instance, can refer to either *crude ore* or *usable ore*. *Crude ore* is often used for *run-of-mine* or *pithead output material* that needs further processing to become a saleable product; *useable ore* may refer to either *high-grade direct-shipping ore*, or to *finished (beneficiated) ore* that has undergone further processing to turn it into a saleable product, such as *concentrate* or *pellets*. Without a MB-consistent system definition, there is a risk for calculation errors with potentially far-reaching consequences: the U.S. World iron ore production statistics between 2000 and 2014, for instance, overestimated the global production of useable ore by 10 to 32%^197^ because Chinese production numbers were interpreted as useable ore, although they actually reported crude ore.

Such misunderstandings can be avoided by publishing material system diagrams or material flowcharts that define key terms and use explicit *reference points* to place reported mine production data into a systems context (Figure 3c). Indeed, the Norwegian Directorate of Mining (DMF) recommends material flowsheets as part of permitting procedures for mining activities.^198^ Similarly, the Canadian Institute of Mining (CIM) notes that material flow diagrams are of “great assistance” for reconciling long-term models with plant production data,^199^ and that mass balances of the major flows should be included for internal as well as public reporting of minerals projects.^199,200^ These guidelines are a first step toward, but not sufficient for, systematic physical monitoring and accounting. They acknowledge that material system diagrams are helpful but do not make their use mandatory, and do not discuss how explicit system boundaries, reference points, and MB principles can facilitate transparent regular (e.g., annual) reporting and monitoring of mine production flows.

## 5 Geomodeling of Material Stocks and Flows

### 5.1 Geomodels for Stock Accounting and Resource Classification

**Figure 4:**
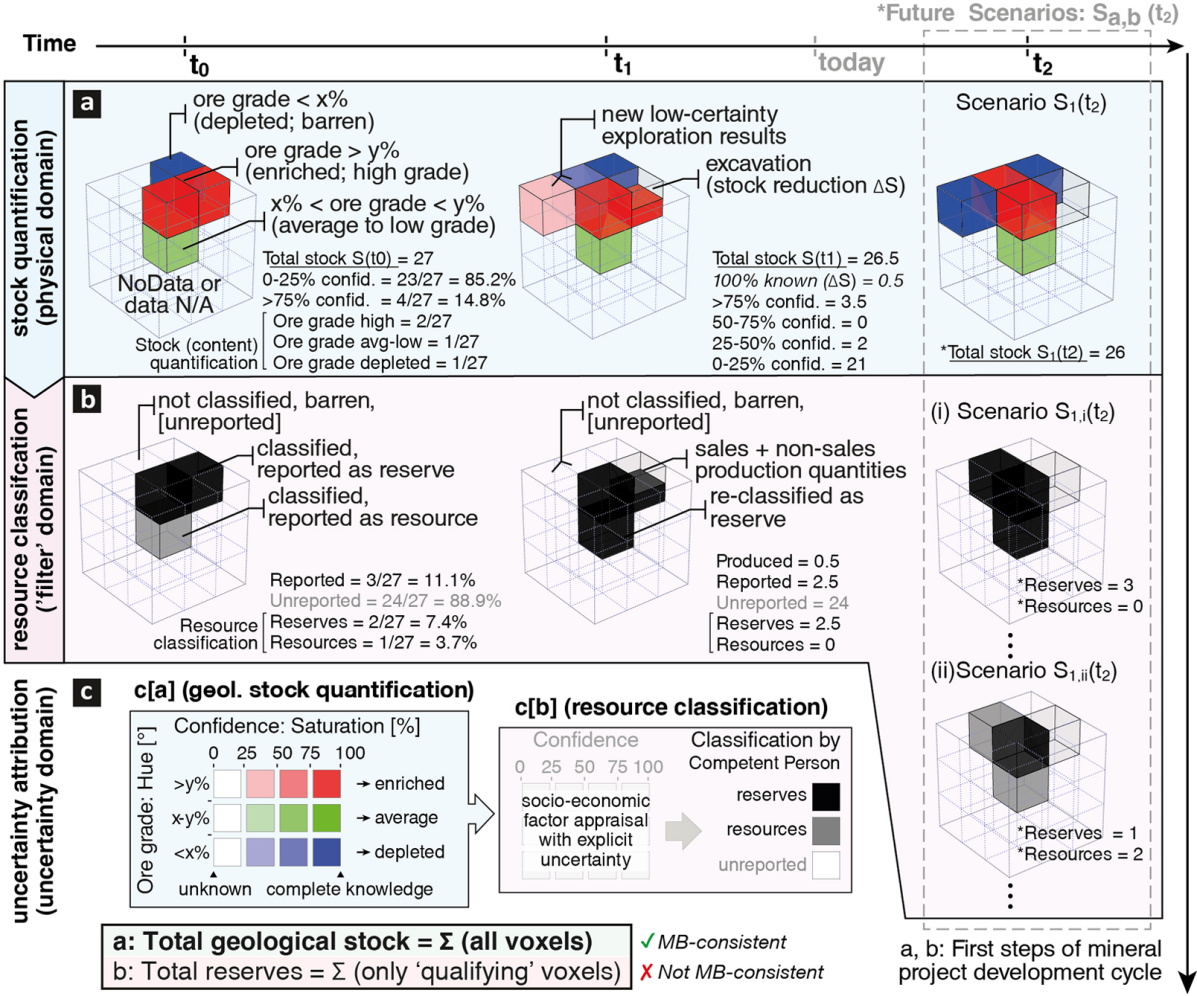
Multidimensional geological stock accounting illustrated as a cube with 27 voxels at three reference points (t0, t1, t2). (a) Geological stock accounting monitors changes of the *physical domain* over time and shows historical extraction as a measured reduction of the total stock S by 0.5 voxels from 27 → 26.5 during t0 → t1 and anticipated further reduction 26.5 → 26 during t1 → t2, assuming stock scenario S_1_. Exploration activity changes only the attributes (e.g., ore grade) and associated uncertainty of the geological stock characterization (2 voxels from 0% → 25–50% confidence during t0 → t1, and from 25 to 50% →>75% during t1 → t2, assuming stock scenario S_1_). (b) Resource classification acts as a *filter domain* that selectively appraises parts of the geological stock to report reserves and resources, while omitting the rest of the geological stock including known but low grade (barren) voxels; Individual geological stock voxels may remain physically unchanged but may nevertheless be reclassified as time passes (1 resources to 1 reserves during t0 → t1) or vice versa (1 reserves to 1 resources during t1 → t2 assuming resource classification scenario S_1,ii_(t2)). (c) Uncertainty attribution is considered as two separate steps: step c[a] addresses solely the uncertainty of the physical attributes for stock quantification; step c[b] incorporates the additional uncertainty of socioeconomic assumptions of resource classification. Color hue (red, green, blue) represents three ore grade classes relative to average crustal abundance (depleted, average to low grade, enriched); color *saturation* (0–25, 25–50, 50–75, 75–100) shows the confidence in the results (unknown to complete knowledge). MB, mass-balance.

Geomodels are digital representations of the Earth’s subsurface^201^ that are essential for addressing a wide range of societal issues.^202^ They can be expressed though gridded volume elements (voxels) and attributes that characterize and quantify continuous physical phenomena such as geological formations, groundwater flows, and other subsurface features.^203^ Geomodels have been extensively used in petroleum reservoir engineering since computers became available in the late 1960s.^88^ Given our previous definition, *geological stocks* can be modeled with voxels and analyzed either as a whole or in parts to quantify the total material content together with its average composition and/or that of selected individual voxels, elements, or substances, for any specific point of time, with a certain level of confidence. This may, for instance, be used to calculate the elementary stock of pure copper in tonnes based on the copper grade distribution within a defined volume, or to quantify the total stock of sand and gravel in a region as the sum of sand-containing voxels, for a specific *reference point* of time. An unlimited number of voxel attributes can be defined to describe stock characteristics. Here, we illustrate *geological stock accounting* in Figure 4a, using only ore grade and uncertainty. The total extraction flow during the time interval from the initial reference state at t0 to a specified reference state at t1 corresponds to an observed stock reduction. Using prospective geomodeling, further stock reduction may be simulated for a future state t2, subject to probabilistic geomodeling, mine design, and operational planning.^204,205^ The extracted material leaves the geological stock subsystem (geosphere) and enters the economy. Notably, natural processes such as erosion move material only within the geological subsystem and do not register as a transfer of material from the geosphere. We deliberately separate the geological stock accounting step (a) from the resource classification (b). Geological stock accounting is necessary to build a robust and MB-consistent full-coverage digital model of the physical reality. Resource classification is conceptualized as an optional and independent additional step that acts as a “filter” to selectively appraise specific stock segments that are thought to be of particular interest for further mineral project development. While we postulate that a MB-consistent geological stock model can always serve as a robust information source for subsequent resource classification and aggregation, earlier sections have outlined that the inverse is impossible: reserve numbers cannot be converted to geospatial models and thus have limited utility for mineral depletion, environmental, and sustainability monitoring and assessments.

### 5.2 Model Uncertainty

Uncertainty is pertinent to the quantification, and visualization of 3D geodata.^206−208^ Epistemic uncertainty arises from incomplete knowledge and can be reduced though new exploration, geological mapping, drilling, and sample analysis.^209,210^ MB-consistent geological stock accounting presumes that the model’s system boundary (i.e., envelope of all 27 voxels in Figure 4) remains fixed though time. This enables spatially explicit uncertainty attribution for every voxel to capture the evolution of knowledge over time (confidence intervals in Figure 4c). Exploration activity is not considered to be a physical material flow and does not affect the system boundary or the total geological stock volume. Rather, new observations reduce the uncertainty of the attributed physical characteristics of voxels (i.e., increase the confidence in the stock characterization). Conversely, measurements during production can provide a “closed-loop”^133^ feedback to validate or reconcile the model and increase the confidence for the remaining in situ material. Both integration of (ex-ante) exploration data and closed-loop (ex-post) analysis and feedback thus make the geological stock model for the remaining stock more accurate, useful, and valuable over time. Reported mineral reserve numbers, in contrast, are valid only at a specific point of time; they have additional uncertainty due to extrinsic socioeconomic assumptions that are difficult to constrain and predict.^211,212^ This shortcoming underscores the strategic benefit of allocating research and funding for MB-consistent geological stock accounting and material flow monitoring: information on material systems describes observable real-world phenomena, helps understand physical changes such as resource depletion, and can contribute to building a continuously growing, versatile, robust, and increasingly accurate global geoscientific knowledge base.

## 6 Framework for Systems Integration

While the credo of the extractive industry has long been “if we can’t grow it, we have to mine it”,^213,214^ one may add *‘but we need robust material stock and flow models to know when, where and how to best get it’*. The clean energy transition, for instance, requires batteries, solar cells, and wind turbines, but national policies seldom quantify how much lithium, indium and dysprosium will be needed to produce them, and where and how to sustainably source the required minerals, components, or products.^215^ Answering these questions is challenging without reliable geospatial information and robust scenario models, which again require systematic mine-site-scale material stock (key issue one) and material flow data (issue two). We argue that a more physical-accounting-centric approach to industry-government data integration is necessary and mutually beneficial.

### 6.1 The Current Situation: Data Fragmentation and Limited Coordination (Figure 5a)

The mining industry collects detailed 3D geological as well as material flow data for site-specific project planning and operational management. However, these data sets are typically stored in proprietary company data “silos”,^216^ and generally not part of public disclosure or formal government reporting (5a, information flow ‘A’). Often, only stock-market-listed mining and exploration companies have public disclosure routines in place. Even in these cases, published data are incompletely georeferenced and lack comparability and consistency both between entities in the same industry and across jurisdictions.^52,114^ Moreover, details on intrinsic physical properties for systematic quantification and characterization of relevant material stocks and flows (e.g., mass and volume of waste rock, whole-rock composition, mineralogy, pH of tailings) are commonly missing. Notwithstanding, data providers such as S&P Global or Wood Mackenzie compile comprehensive datasets from the available company data; governments, on the other hand, rarely systematically harvest these data to supplement or validate their own information.

**Figure 5:**
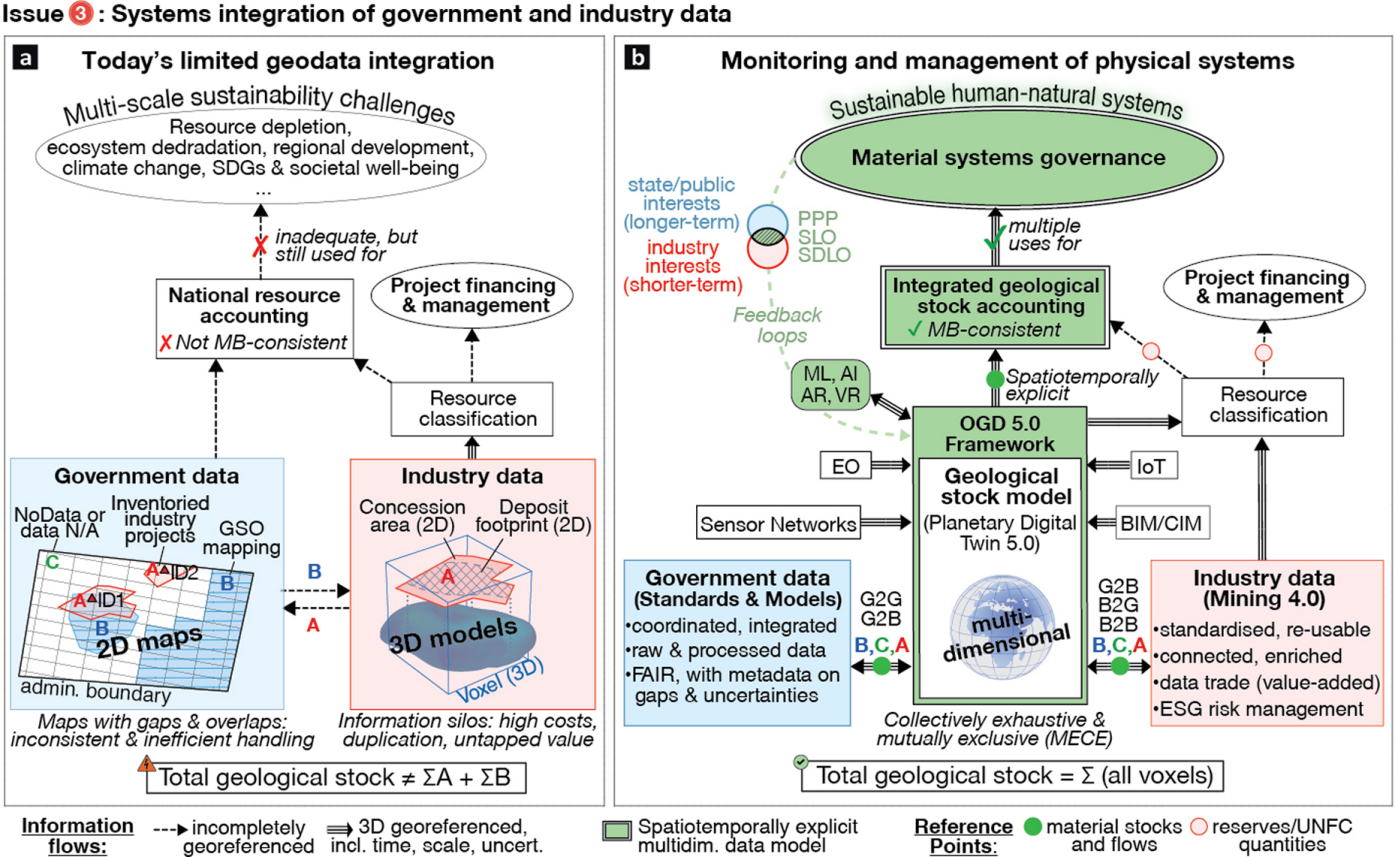
(a) Today’s information flows on nonrenewable mineral resources result in incomplete, fragmented, and inconsistent knowledge that is unsuitable for addressing systemic issues related to sustainable resource management. (b) The proposed monitoring of physical systems is based on an Open Government Data (OGD) framework that supports multidimensional geodata integration, mass-balance (MB) consistent geological stock accounting, and spatiotemporally explicit material systems governance. PPP: Public-Private Partnership; SLO: Social License to Operate; SDLO: Sustainable Development License to Operate; GSO: Geological Survey Organization; EO: Earth Observation; IoT: Internet of Things; BIM/CIM: Building/City Information Modeling; ML: Machine Learning; AI Artificial Intelligence; AR/VR: Augmented/Virtual Reality; G2B, G2G, B2B, B2G: Government-to-Business data sharing, etc.

Governments collect data by different means and manage enormous amounts of multiscale geospatial information. Data provided by geological survey organisations (GSOs), for instance, are sought-after for mineral exploration and project planning (“information flow” arrow ‘B’), and are key drivers for economic development.^217−220^ In Western Australia, for instance, GSO data delivered an estimated 31-fold total return on investment.^219^ However, the minerals and mining-related datasets are still mostly 2D, have coarse resolution, and mainly cover areas where publicly funded exploration campaigns have been conducted (“mapped areas” labeled ‘B’). National mineral inventories and mineral statistics combine such government data with mandatory company reporting, and occasionally also voluntary public disclosures and information from industry associations and commercial data providers.^41,221^ Such data compilation and integration from sources that are poorly standardized, diverse, and can only be selectively sampled is time-consuming and costly.^42^ Moreover, nongeospatial “external” data cannot readily be integrated into existing geomodels, but rather only indirectly linked to administrative records (e.g., mapped concession areas ‘A’) or approximate locations of production sites (e.g., ‘ID1’). This entails that national mineral inventories have known and unknown (hidden) spatiotemporal data gaps (e.g., irregularly updated reserves information; areas without geodata ‘C’, NoData or data N/A), irreconcilable data overlaps (e.g., contradictory government-industry geodata), and thematic inconsistencies (e.g., different survey methods, reporting dates, or spatiotemporal resolution). Poor metadata and lack of documentation on data collection, cleaning, and aggregation workflows further increases uncertainty. Altogether, today’s government-industry data integration produces inconsistent 2D maps with hidden gaps and unnecessary overlaps. This results in national spatiotemporal data coverages that are neither *mutually exclusive (A∩B∩C:= 0)*, nor *collectively exhaustive (total geological stock:= ΣA + ΣB + ΣC)*.

### 6.2 Facilitating Integrated Monitoring of Physical Systems (Figure 5b)

Interdisciplinary, cross-scale, digital Earth science platforms and information infrastructures are needed for environmental monitoring, Big Data analytics and cross-disciplinary Earth science.^222^ Such platforms could form the basis for hybrid modeling approaches^140^ that obey physical laws, while leveraging data-driven machine learning to better understand the Human–Earth system.^223^ The OneGeology initiative, for instance, was established to harmonize global geoscience data,^224^ while the EarthServer community wishes to allow users to “ask any question, any time, on any volume”.^225^ MB-consistent geological stock accounting aligns with these visions, with Figure 5b showing its role as part of what we call the *monitoring of physical systems*. Advances in three key domains are particularly favorable for further developments:

(i) *Earth Observation (EO) and Geomodeling*. Earth observation (EO) continuously expands our knowledge of an urbanizing planet^226−228^ with exponentially increasing amounts of global-scale, multidimensional time-series data. Data acquisition technologies such as satellites and drones that interact the Internet of Things (IoT) facilitate both global mapping of mining land use,^229^ and high-resolution mine-site-scale monitoring of production stockpiles and tailings storage facilities.^230,231^ Such remote and in situ measurements are key to the extractive industry’s *Mining 4.0* vision of smart and connected digital transformation.^232,233^ It is estimated that 95% of EO data have never been accessed, partly due to challenges with managing its volume, variety, veracity, velocity, and the difficulty to extract value (the five Vs).^222^ This indicates that there is a huge potential for Big Earth Data fusion,^222^ geospatial artificial intelligence (GeoAI),^234^ and cloud-based computing, which together can help improve data accessibility and support investigative approaches also for users with limited knowledge.^235,236^ Simultaneously, free or relatively inexpensive access to open government servers^223^ or proprietary platforms such as Google’s Earth Engine^237^ and Microsoft’s Planetary Computer,^238^ coupled with geodata modeling environments including the Open Data Cube (ODC)^236,239^ and advances in data processing^240^ and visualization technologies,^241−243^ facilitate large-area high-resolution geomodeling.^244−246^ Digital twins^247,248^ may soon become standard tools for modeling the geological subsurface together with production facilities at mine-site (plant) scale, and may be part of larger models that integrate geological information with urban-scale building- and city information models (BIM/CIM) into regional GeoBIM systems.^249,250^ Indeed, two decades after the former Vice President of the USA Al Gore outlined his vision of a “Digital Earth”,^251^ the UN-led Coalition for Digital Environmental Sustainability^252^ has recently declared the development of a “Planetary Digital Twin” a strategic priority for the sustainability transformation. Given the accelerating rate of innovation, we can imagine multidimensional (e.g., 6D = *x*,*y*,*z* + time + scale/resolution + uncertainty)^253,254^ Digital Earth Science Platforms^254−256^ that allow us to model historical, monitor ongoing, and simulate future geological and anthropogenic stock changes and material flows through space and time.
(ii) *Multidimensional Geoinformation Management*. The value of data is maximized by reuse.^257^ Standards and protocols such as the forthcoming ISO 19123-1 on multidimensional “coverages”^256^ and the “Spatial Data on the Web Best Practices”^258^ facilitate sharing and integration of georeferenced multidimensional data with their original granularity (triple-lined arrows). Standardization can be voluntary or mandatory: the European INSPIRE Directive on establishing an infrastructure for spatial information,^259^ for instance, defines legally binding goals for geodata harmonization across European countries, while the International Union of Geological Sciences follows a voluntary “Big Science Initiative” standardization approach.^260,261^ Development of a multidimensional “Open Government Data (OGD5.0) Framework for Physical Accounting” can draw on such efforts (cf. Figures 4, 5), while spatiotemporally explicit and MB-consistent reporting can support *mutually exclusive* and *collectively exhaustive*(119) data integration and the establishment of digital twins and “cyber-physical systems”.^262^ Multistakeholder involvement and Public-Private Partnerships (PPPs)^263^ can commit to “co-create”^264^ the OGD5.0 for secure, consistent, and integrated Government-to-Government (G2G) and Government-to-Business (G2B) information exchange.^218,265^ For governments, which serve as stewards for data and natural resources on behalf of society, a material systems approach can help close data gaps, reduce industry-government information asymmetries, and build public knowledge capital to support long-term sustainable development. The industry can benefit from access to previously unavailable information through the B2B data trade. This would allow partners to exploit the collective data volume though machine learning (ML), artificial intelligence (AI),^234,266,267^ and digital laboratories with augmented and virtual reality (AR/VR),^268,269^ and can inform mineral systems analysis^270^ and exploration,^47,217^ process innovation,^266^ and supply chain management.^103^ Similarly, transdisciplinary stakeholder collaborations^271^ can contribute to joint problem solving.
(iii) *Policy Trends and Best Practice Examples*. Knowledge sharing between government and industry, and across supply chains, is a key challenge for mineral resource governance.^156,272^ The FAIR^273^ and OGD^274^ principles, OECD Recommendations,^275^ and the Integrated Geospatial Information^276^ and Global Statistical Geospatial Frameworks^277^ provide high-level guidance for addressing “data and organizational silos”.^278^ However, additional efforts are needed to ensure more effective data collection (e.g., to avoid data duplication and target key gaps), facilitate better data integration (e.g., georeferencing, MFA system diagrams/flowsheets with explicit data reference points), and promote data reuse (e.g., FAIR principles, PPPs). Various studies have found that voluntary reporting commitments by mining companies emphasized documentation of compliance over actual data disclosure,^159,279^ failed to guarantee timely and granular project-by-project level reporting,^280−282^ and had limited impact on mine-site level action.^148,279^ In response, governments are called upon to use their legislative, regulatory, and policy tools to implement new frameworks that support systematic ESG reporting (cf. S2)^53,56,283^ and granular data disclosure.^194,283−288^ Governments could use a common physical systems approach to monitor and manage material systems, and to set predictable but yet flexible framework conditions^263^ that allow the extractive industries to compete with their best capabilities for securing future mineral supply. By inviting/requiring mining and exploration companies to submit collected geodata into secure public databases, long-term public knowledge and value creation can be maximized.^160^ MB-consistent monitoring can promote transparency (e.g., materials certification, traceability) that helps build public trust, contributes to fighting theft, corruption, and tax fraud (e.g., fraudulent transfer pricing) and can ensure that mining activities achieve their project-specific commercial interests, while fulfilling their broader societal obligations toward the SDGs.^94,146,147^

Altogether, we can maximize the robustness and value of reported material stock and flow data by ensuring that they are (a) georeferenced and MB-consistent over consecutive accounting steps, which enables geospatial analytics, bottom-up raw material analysis, and scenario development with MFA;^289^ (b) collectively exhaustive regarding spatial coverage and stakeholders including SMEs,^283^ which facilitates more representative aggregation across project, enterprise, and jurisdictional levels; and (c) open (FAIR), which supports SLO and SDLO negotiations and digital innovation. Reported site-scale data can be used in sustainability assessments^55^ to inform investors about project risks and opportunities,^191,290,291^ and to identify trade-offs and synergies across different projects. Overall, MB-consistent data on physical stocks and flows can help to understand decision path dependency^292^ (e.g., historical mine production data allow approximation of accumulated mine waste stocks), and help set science-based targets^293^ for mineral supply within the “sustainability solution space”^294^ or “safe operating space”.^295^ Future efforts toward integration can draw on experiences from following three initiatives:

(1) the *European Open Data Directive*, which requires from its member States that “public sector bodies and public undertakings shall make their documents available [...] in formats that are open, machine-readable, accessible, findable, and re-usable [...] at the best level of precision and granularity”.^296^ Six thematic categories of *high-value data sets* are highlighted: geospatial, Earth observation and environment, meteorological, statistical, company information and ownership, and mobility.^297^ Moreover, the European Commission announced in its *European strategy for data*(167) that it will explore a regulatory framework to govern the public sector’s reuse of privately held data of public interest, and will launch a strategic “*Destination Earth*” initiative to develop a very high precision digital model of the Earth.
(2) the Dutch law on subsurface information, which establishes the *Dutch National Key Registry of the Subsurface* (BRO) as a central data repository to collect, store, and manage all publicly funded subsurface data.^298^ A crucial aspect of the BRO is that it integrates confidential personal and industry information related to licensing and use, and that its stepwise implementation is intended to ultimately include data on all subsurface construction activities including measurements related to exploration, extraction, and storage of minerals and geothermal heat.
(3) the *Norwegian National Data Repository for petroleum exploration and production data* (Diskos), which is a public-private partnership established in 1992 as a joint venture between the Norwegian government and the oil companies on the Norwegian Continental Shelf.^299^ Diskos ensures secure, efficient, and standardized data management on behalf of its members, with shared overheads and added benefits. The system holds all the data of all licensees including detailed project metrics (i.e., all geological data, time-based forecasts, investment and operating cost schedules, production, emissions, cash flows etc.).^300^ This reduces individual data handling costs as company repositories are no longer required, allows business-to-business (B2B) trade of entitlements to confidential data, and facilitates business-to-government (B2G) reporting. Although company data remain confidential, they are accessible for authorized government processes. This decreases the reporting burden, expedites processing, and reduces administrative costs because the government already has access to the information it requires for taxation and resource governance. Diskos also incorporates the information that financial regulators typically require for stock market disclosure, which instills confidence, promotes transparency, and ensures consistency between industry reporting and government inventories. By leveraging the “digital economy”^268^ for exploration and minerals development,^301^ common repositories can stimulate data reuse, value maximation in mining, and more transparent taxation. Finally, Diskos contributes significantly to expanding Norway’s collective knowledge capital as new data on licensed and unlicensed areas are continuously integrated. This information will eventually be made public as the needs for confidentiality cease or when licenses expire or are relinquished.

All three initiatives make some level of stakeholder coordination and reporting mandatory. They maximize collective value generation from both a business and societal perspective, clarify roles and responsibilities, and advocate data sharing and reuse.

## 7 Implementing Physical Monitoring

Transdisciplinary^271^ research and coordinated efforts can help to ensure that (1) reported data on stocks and stock changes are explicitly georeferenced in space and time, and that documentation includes the original granular data on volume, mass, composition, and relevant intrinsic material properties; (2) reporting mandates cover all relevant stakeholders (including both the formal sector and estimates on artisanal mining) and all relevant material stocks and flows for calculating mass balances across processes; and that (3) reporting workflows use common data standards, MFA system diagrams with explicit reference points and terminology, and multidimensional geodata models that maintain MB-consistency across processing stages through space and time.

Given the increasing momentum toward sustainability reporting, the global appetite for transformative change, and the emergence of Big Earth Data technologies, MB-consistent physical accounting is becoming feasible. Better industry-government coordination and data integration are of mutual benefit, supporting the statement that “without a common framework to organize findings, isolated knowledge does not cumulate.”^302^ Governments and industry typically share the risks and rewards of mineral extraction in the monetary economy (e.g., operating surplus, taxes), physical economy (mining waste, material supply), and digital economy (data waste, data reuse). Our definitions and framework for MB-consistent geological stock accounting are designed to guide efforts toward an integration of terminology and data, and sustainable management of human-natural physical systems. For implementation, we suggest the following next steps:

(i) *Review and Adapt Policy Frameworks and Legislation for Physical Accounting*. Intergovernmental bodies and governments can review current mineral resource, mine production, and ESG reporting to identify their key gaps and limitations with focus on geodata integration and material stock and flow analysis. To clarify information under their stewardship, they may use their platforms to showcase typical applications and limitations of current data and outline key benefits of mass-balance-consistent accounting. Next steps may include defining roles and responsibilities across stakeholders to formalize data sharing and standardization; assigning explicit mandates to address data fragmentation and promote cross-institutional integration; enacting new policies for systematic monitoring of the physical human-natural system; and developing data-driven scenario models to inform decision-making. International partners may include the UN Statistics Division (UNSD), International Resource Panel (IRP), UNECE Expert Group on Resource Management (EGRM), UN Initiative on Global Geospatial Information Management (UN-GGIM), and UN-led Coalition for Digital Environmental Sustainability (CODES). On a country-level, relevant bodies include GSOs, mining directorates, mapping and planning authorities, environment agencies, and statistical offices, as well as professional associations, NGOs, academia, and industry.
(ii) *Develop Infrastructures for Multidimensional Geoinformation*. Through transdisciplinary government mandates and partnerships, appointed agencies and relevant stakeholders can review how technical data standards, reporting workflows and accounting systems (e.g., ISO,^256^ INSPIRE,^259^ UNFC,^116^ SEEA,^145^ UNEP^194^) may be adapted to facilitate systematic and granular disclosure in-line with *OGD*, *FAIR*, and *SDLO* principles, and how to automate consistent integration for multidimensional minerals-related material stock and flow information. A first step toward promoting research and development of technical infrastructures could make it mandatory for companies and data providers to map their current reporting of materials-related stock and flow data using MFA system diagrams (flowsheets), standard terminology, and explicit reference points. Funding bodies and relevant stakeholders may consider pilot projects to evaluate this idea, define and map relevant terms, and initiate the development of common data models for physical monitoring, multiscale modeling, and MB-consistent accounting.

## 8 Conclusions

Finding new ways to understand stock-service-benefit relations and make human interactions with the natural environment more sustainable is a key challenge for Earth System Science.^140,141,303,304^ The proposed definition and conceptual approach combines multidimensional geomodeling with MFA to facilitate mass-balance-consistent geological stock accounting as part of efforts to secure a sustainable mineral supply within biophysical planetary boundaries.^66,169,305,306^ It marks a paradigm shift for mineral resource governance by (1) enabling integrated monitoring of stock changes and impacts over time; (2) creating a robust basis for on-the-fly calculation of geological and waste stocks, mineral reserves, ESG risks, and asset portfolios; (3) pooling of government-industry information for mineral systems analysis, predictive mapping, and spatial planning to “safeguard” geological deposits for future mining; and by (4) using MFA to inform strategies for mineral supply, circular economy, and on how to balance sustainability trade-offs. While legal and proprietary issues, coordination, and the need to change legacy data systems pose a sizable challenge, investments into integrated physical monitoring and modeling are likely to yield substantial long-term benefits. Data fragmentation, ownership rights to commercial-in-confidence information, as well as diverse definitions, reporting schemes, and institutional responsibilities call for more research on how to standardize terminology, streamline reporting requirements, and collaborate to make mineral information more available and useful for resource management. We expect that industry and professional associations, regulators, and relevant government and international agencies can start with small first steps to promote standardized and granular reporting of site-scale geological stock and material flow data without imposing significant additional burdens on operators, as much of the relevant data are already routinely collected. Further research could help to better understand how the growing momentum of Earth systems monitoring, digital twins, and multistakeholder resource governance dialogue can be combined with FAIR data policies and sustainability efforts to accelerate the buildup of geoscientific knowledge of the “knowns” (showing, e.g., that Europe is not necessarily resource-poor), to better inform efforts on “what needs to be known” (e.g., where to target exploration and direct innovation) for the global public good.

*Resource realists* play a vital role in research on methods and models to monitor and anticipate physical human-nature interactions, can support initiatives that build the common global Earth System knowledge base, and can help communicate the interconnected challenges of mineral resource depletion and sustainable supply to explore new pathways for satisfying our societal needs within planetary boundaries.

## Supporting Information Available

Materials and methods section describing literature selection and bibliometric analysis; timeline of historical events with reference list; notes on mass balance consistency in financial reporting and the UN System of Environmental-Economic Accounting (PDF)
