# Supplementary material for: Mass-Balance-Consistent Geological Stock Accounting: A New Approach toward Sustainable Management of Mineral Resources
Source: Environ Sci Technol. 2024 Jan 2;58(2):971–90. doi: 10.1021/acs.est.3c03088 (PMC10795188; doi:10.1021/acs.est.3c03088)
Supplement: Supplementary file 1 — es3c03088_si_001.pdf [file es3c03088_si_001.pdf]

Supporting Information

Mass-balance-consistent geological stock accounting – a new approach towards sustainable management of mineral resources

Mark U. Simoni[1,2], Johannes A. Drielsma[3], Magnus Ericsson[4], Andrew G. Gunn[5], Sigurd Heiberg[6], Tom A. Heldal[1], Nedal T. Nassar[7], Evi Petavratzi[5], Daniel B. Müller[2]*

[1] Geological Survey of Norway, Leiv Eirikssons vei 39, 7040 Trondheim, Norway

[2] Norwegian University of Science and Technology, Industrial Ecology Programme, Høgskoleringen 5, NO-7034 Trondheim, Norway

[3] Drielsma Resources Europe, 2585 GT The Hague, Netherlands

[4] Luleå University of Technology, Department of Business Administration, Technology and Social Sciences, 97187 Luleå, Sweden

[5] British Geological Survey, Keyworth, Nottingham, NG12 5GG, United Kingdom

[6] Petronavit AS, C/o Heiberg, Stokkahagen 23, 4022 Stavanger, Norway

[7] U.S. Geological Survey, National Mineral Information Center, 12201 Sunrise Valley Dr., MS 988, Reston, VA 20192, USA

* Corresponding author: Mark Simoni, mark.simoni@ngu.no, +4746777449

Contents

S1. Materials and methods 2
S1.1. Literature selection 2
S1.2. Bibliometric analysis of mass-balance-consistent accounting for minerals 3
S1.3. Historical development of MFA 4
S2. Timeline of resource classification and sustainability efforts 7
S3. Resources and reserves information in financial industry reporting 10
S4. MFA and the System of Environmental-Economic Accounting 11
S5. References: 14

S1. Materials and methods

Key reviews from the domains of economic geology, industrial ecology, and Earth systems modeling highlight the urgent need for transdisciplinary systems integration. Prior review articles from industrial ecology, for instance, are testament to the growing recognition of material flow analysis (MFA) as a robust tool for quantitative assessments and scenario modeling. In Earth and environmental science, the quantification of natural processes, substance flows, and elemental cycles (e.g. erosion and deposition, ore deposit formation, global carbon cycle) has long been central for understanding the Earth system and its spheres. However, it is still debated how mineral resources can be robustly quantified and monitored. Here, we review how mass-balance (MB) principles may support integration of the anthropogenic and the geological subsystems into physical Earth System models, and how MB-consistent geological stock accounting can facilitate scenario modeling and sustainability analysis. Rather than aiming for a complete and systematic literature review of one specific research domain, our Critical Review article takes a broader approach to provide a transdisciplinary and integrative overview across the relevant fields that together define our topic, as shown in Figure S 1-1.

S1.1. Literature selection

We queried the Web of Science (WOS), Scopus, and Google Scholar with various search term combinations including "(environment* OR sustainab*) AND (mining OR mines OR ore OR (mineral reserves) OR (mineral resources) OR Deposits)", "material flow analysis", "geological stocks", "mineral resource depletion", "multidimensional geomodels", "digital twin", and others to collect relevant publications for an integrative literature review approach[1] that can shed light on key terminology, important concepts, and critical knowledge gaps across different research domains. Moreover, we evaluated the references in individual publications that we considered particularly relevant to find further research to include. Next to scientific articles, we also considered documents from the 'grey literature' domain, including books, industry standards, international agreements, and government reports, as these relate to current public administration and business practices that are often not covered by the formal literature, but nevertheless relevant for physical accounting. Queries of WOS and Scopus produce broadly overlapping but also partially complementary results. We used published workflows[2,3] to combine the two datasets into a more complete collection for further analysis and discussion.

S1.2. Bibliometric analysis of mass-balance-consistent accounting for minerals

For our bibliometric analysis and visualization of results, we used WOS, Scopus, and Bibliometrix.[4] Publications in the grey literature domain are not indexed in WOS and Scopus and were thus not part of the Bibliometrix analysis. Figure S 1-1 (a) shows the top 10 author keywords of the cited corpus of publications, illustrating the broad thematic coverage of our review. Notably, the figure underrepresents the topic of mineral resource classification and accounting, which is largely discussed outside the formal literature. Figure S 1-1(b) emphasizes the importance and the broader context of geological stocks serving as the ultimate source of all mineral raw materials in the anthroposphere. Our *'Mineral Materials Tree'* highlights that a robust understanding and mass-balance-consistent monitoring of geological stocks and stock changes is critical for strategic materials management. It shows that mass balances may be used to resolve the complex linkages and trade-offs involved in the extraction and use of minerals, and that physical monitoring can be facilitated by digital technologies, knowledge integration, and holistic governance approaches.

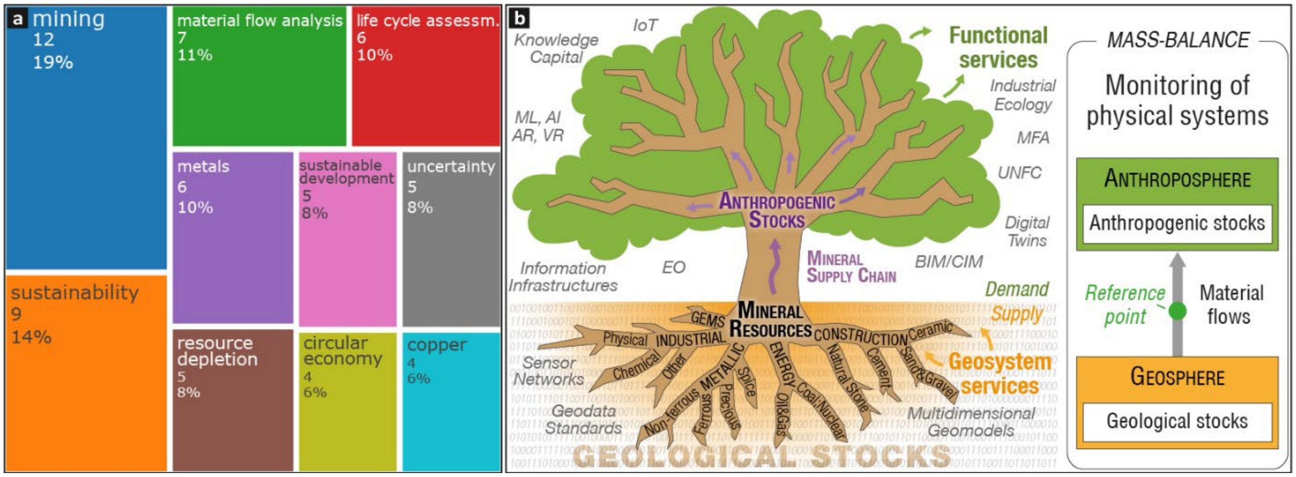

Figure S 1-1: (a; left) Bibliometric analysis of top-ranked author keywords in publications we reviewed, showing the broad thematic coverage of our article. Represented are only keywords from articles that are indexed by WOS or Scopus (149 of 296), which excludes grey literature. (b; right): Table of contents figure (TOC) of our article. The 'Mineral Materials Tree' symbolizes the vital role of geological stocks as hosts of the main mineral resource groups that are mined to sustain the global non-renewable raw materials supply. The physical systems perspective also covers key elements of data-, knowledge-, and systems integration, which are reviewed and synthesized in the integration and implementation sections of our article.

Analyzing the trend topics in author keywords in our corpus of surveyed literature over the past decade (Figure S 1-2) shows that material flow analysis (MFA) has become a hot topic in recent years.

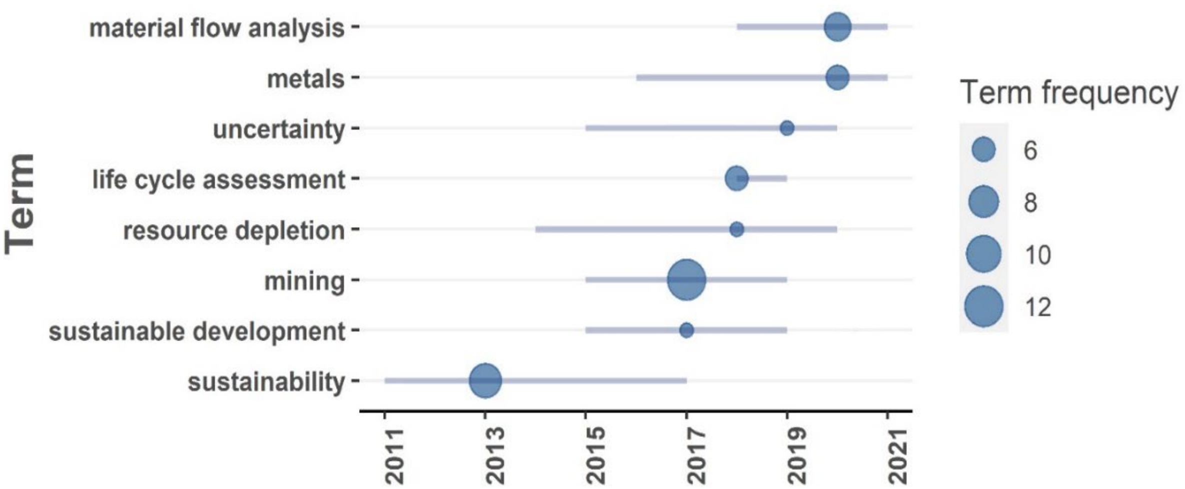

Figure S 1-2: Evolution of trend topics in author keywords of the surveyed literature, 2011-2021.

S1.3. Historical development of MFA

We used Google Scholar to compile a systematic and broad overview over the total number of publications linking “material flow analysis” with “mineral reserves”. The combined query “material flow analysis” – [i.e., NOT/without the term] “mineral reserves” for the period 1970 to 2022 shows strong growth in the adoption of MFA outside of the minerals community, with publications in this group accounting for 99.1% of all publications mentioning MFA (Figure S 1-3). In contrast, publications that mention both terms “material flow analysis” + “mineral reserves” (i.e., the mutually exclusive complement of the query above) only make up 0.9%, or 223 out of a total of 24048 publications. That the annual number of publications mentioning both “material flow analysis” + “mineral reserves” remained stable at around 20-30 per year over the past 10 years indicates that MFA has not been widely adopted by the geological and minerals community, while interest in MFA outside of the geological community has rapidly grown. This highlights the gap that our Critical Review article addresses. For reference, we also plot the trends for “3D geological model” and “mineral resource depletion”.

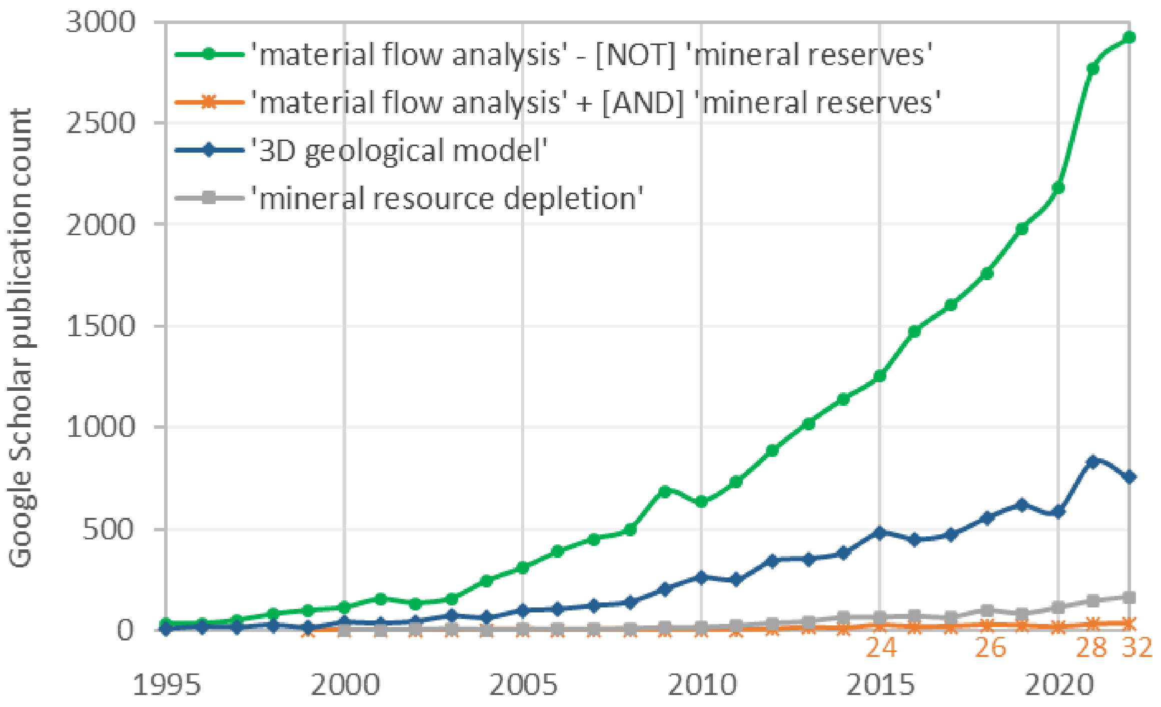

Figure S 1-3: Google Scholar-indexed annual publication count for the combined query "material flow analysis", explicitly excluding (-) and including (+) "mineral reserves"; also shown are "3D geological model" and "mineral resource depletion".

The relative importance of MFA in the field of geosciences versus in the field of environmental sciences and engineering can be compared using WOS to calculate the annual field- or category-normalized research output.[5,6] WOS uses a journal-based classification system where journals are assigned to one or more of around 250 categories.[6] We normalize the number of publications for an "All fields" query "material flow analysis" for two mutually exclusive and complementary WOS Category (WC) combinations (Figure S 1-4): One, "WC=(Environmental OR Engineering NOT Geosciences, Multidisciplinary NOT Geology NOT Mining Mineral Processing NOT Geochemistry)" and two, "WC=(Geosciences, Multidisciplinary OR Geology OR Mining Mineral Processing OR Geochemistry NOT Environmental NOT Engineering)".

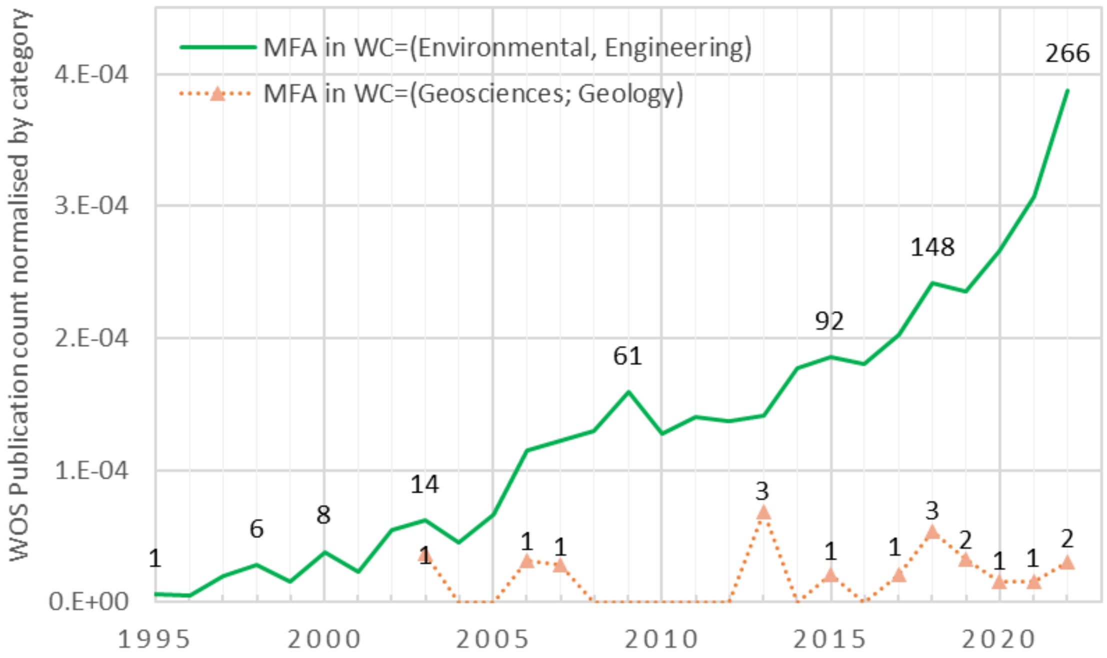

Figure S 1-4: Relative importance of "material flow analysis" (MFA) calculated per WOS category "Environmental, Engineering" and "Geosciences, Geology", normalized for comparison. Labels show total annual publication count per category, not normalized.

The results confirm our observation from Figure S 1-2 and illustrate that MFA is gaining importance in the fields of environmental science and engineering. In geology and geosciences, in contrast, MFA is barely nascent, as we had previously seen for Figure S 1-3. Altogether, there has been surprisingly little discussion about integrating geological mapping and resource modeling with MFA, despite the latter's proven utility for monitoring and modeling material systems at different scale, from facility to national and global levels (cf. section 2, "A brief review of mass-balance-consistent accounting").

Further research on applying MFA in geosciences, including the development of dynamic and spatiotemporally resolved system models, could provide data and methods to evaluate raw material supply alternatives and address sustainability issues. That mining is inevitable and yet faces increasing challenges, makes robust physical monitoring and analytical methods that support planning even more pertinent.

S2. Timeline of resource classification and sustainability efforts

The history of mining dates to at least 43 000 years BP.[7] Agricola's seminal "De Re Metallica"[8] published in 1556 and the U.S. Geological Survey (USGS) "Principles of a resource/reserve classification for minerals"[9] of 1980 are among many notable contributions that established the methodology and terminology for how to describe, quantify, and classify mineral deposits and their potential for being mined to produce tradable commodities. Many studies on resource classification are published only in the grey literature domain, for instance by geological surveys, industry associations, and UN bodies. While they are typically not covered by literature reviews and bibliometric analyses, we nevertheless consider them relevant for understanding the concepts and the technical documentation behind reported mineral resources and reserves, mineral statistics, and current mineral resource accounting. To close this gap in the scientific discourse, we explicitly include important grey literature in our Critical Review. The concept of sustainability, in turn, has also seen a significant evolution since Von Carlowitz published "Sylvicultura Oeconomica"[10] in 1713. The report "Limits to Growth"[11] by the Club of Rome in 1972 and ensuing discussions have highlighted the importance of quantitative resource assessments for sustainable development. In recent years, mineral resource governance and sustainable mining have taken a more central role in the international debate. Mining companies face increasing pressure to adopt international reporting and sustainability standards. The timeline in Figure S 2-1 and supporting references in Table S 2-1 illustrate that national and international mineral resource classification standards are iteratively revised and increasingly harmonized, both in response to notable events impacting the mining industry including stock market frauds (e.g. Bre-X in 1997)[12], wars,[13] and mine disasters, and to accommodate new accounting concepts, domestic and international raw material policies, and converging global sustainability efforts.

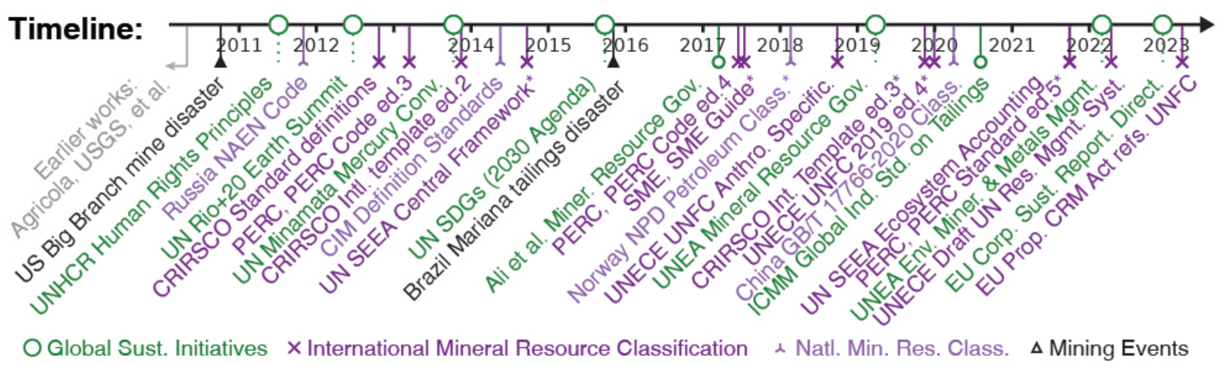

Figure S 2-1: Timeline of international and national mineral resource classification standards alongside global sustainability initiatives and reports, and consequential mining events since 2010. Acronyms: USGS, U.S. Geological Survey; UNHCR, United Nations Human Rights Council; NAEN, National Association of Subsoil Expertise; UN, United Nations; CRIRSCO, Committee for Mineral Reserves International Reporting Standards; PERC, Pan-European Reserves and Resources Reporting Committee; CIM, Canadian Institute of Mining, Metallurgy and Petroleum; SEEA, System of Environmental Economic Accounting; SDGs, Sustainable Development Goals; SME, Society for Mining, Metallurgy & Exploration; NPD, Norwegian Petroleum Directorate; UNECE, United Nations Economic Commission for Europe; UNFC, Framework Classification for Resources; Anthro. Specific., Anthropogenic Specifications; UNEA, United Nations Environment Assembly; GB/T, GuoBiao/TuiJian (Chinese, national standard/recommended); ICMM, International Council on Mining and Metals; EU Corp. Sust. Report. Direct, European Union Corporate Sustainability Reporting Directive; EU Prop. CRM Act, European Union Proposal for a Critical Raw Materials Act.

Table S 2-1: Overview of literature referenced in Figure S 2-1. *Denotes current edition of the respective standard or guidelines. Abbreviations are explained in the references section.

Author	Year	Description	Date	International Classification	National Classification	Sustainability Initiative	Mining Event	Reference Nr.
US	2010	Big Branch mine disaster	05.10.2010				x	-
UNHCR	2011	Human Rights Principles	06.07.2011			x		14
Russia	2011	NAEN Code	31.10.2011		x			15
UN	2012	Rio+20 Earth Summit	22.06.2012			x		16
CRIRSCO	2012	Standard definitions	23.10.2012	x				17
JORC	2012	JORC Code ed. 6*	20.12.2012		x			18
PERC	2013	PERC Code ed. 3	15.03.2013	x				19
UN	2013	Minamata Mercury Convention	10.10.2013			x		20
CRIRSCO	2013	Intl. template ed. 2	15.11.2013	x				21
CIM	2014	Definition Standards	19.05.2014		x			22
UN	2014	SEEA Central Framework*	22.09.2014	x				23
UN	2015	SDGs (2030 Agenda)	25.09.2015			x		24
Brazil	2015	Mariana tailings disaster	05.11.2015				x	-
Ali et al.	2017	Miner. Resource Gov.	16.03.2017			x		25
PERC	2017	PERC Code ed. 4	16.06.2017	x				26
SME	2017	SME Guide*	15.07.2017	x				27
Norway	2018	NPD Petroleum Classification*	20.02.2018		x			28
UNECE	2018	UNFC Anthro. Specific.	28.09.2018	x				29
UNEA	2019	Mineral Resource Gov.	28.03.2019			x		30
CRIRSCO	2019	International Template ed. 3*	15.11.2019	x				31
UNECE	2019	UNFC 2019 ed. 4*	30.12.2019	x				32
China	2020	GB/T 17766-2020 Classification	31.03.2020		x			33
ICMM	2020	Global Ind. Std. on Tailings	05.08.2020			x		34
UN	2021	SEEA Ecosystem Accounting	29.09.2021	x				35
PERC	2021	PERC Standard ed. 5*	01.10.2021	x				36
UNEA	2022	Env. Miner. & Metals Mgmt.	02.03.2022			x		37
UNECE	2022	Draft UN Res. Mgmt. Syst.	14.04.2022	x				38
EU	2022	Corp. Sust. Report. Direct.	14.12.2022			x		39
EU	2023	Prop. CRM Act refs. UNFC	16.03.2023	x				40

S3. Resources and reserves information in financial industry reporting

How much industry information ultimately becomes publicly available for further use varies: many jurisdictions require standardized *financial* reporting,[41] but information on reserves and resources (arguably the key assets of mining companies) is not part of financial statements and remains off-balance-sheet.[42,43] A survey by the International Financial Reporting Standards Foundation found that only 46 of 177 jurisdictions specified the use of a resource classification in local non-financial reporting regulations.[43] The supporting technical assessment reports[44] may contain some useful information for physical accounting.[43] However, few regulators make the systematic public disclosure of these technical reports mandatory. Moreover, the contained information commonly comes in non-geospatial formats and thus is incompletely georeferenced (e.g., pictures of maps in PDF file format, data tables without geospatial coordinates, non-standardized mineral deposit descriptions in text). Documentation lacks comparability and consistency both between entities in the same industry and across jurisdictions.[43,45] In addition, granular public reporting tends to be limited to companies that are listed on the stock markets, often shows only some of their projects, and may not be updated on a regular (e.g., annual) basis. Harmonized and standardized terminology and a common framework for systematic documentation of material stocks and flows including geospatial information may help to address these data gaps, and improve data availability and interoperability. Moreover, technical documentation that builds on a MB-consistent physical accounting approach can make financial disclosures more transparent, and reported data more useful for resource governance.

S4. MFA and the System of Environmental-Economic Accounting

Material flow analysis and the United Nations System of Environmental-Economic Accounting (SEEA)[23] are, by design, based on different principles. MFA as a scientific method is designed to map and model *physical stocks and flows of materials and energy* in, and between, the anthropogenic and natural environment in a MB-consistent manner. MFA formalizes the concept of "physical accounting" though the *principle of conservation of mass*, and (i) typically requires a spatiotemporally explicit geographical system boundary in three dimensions (3D) for the target region of interest; (ii) characterization of the relevant stocks and flows in the different spatial compartment(s) in mutually exclusive and collectively exhaustive (MECE) terms; and (iii) systematic data in physical units to quantify interlinked material stocks and flows in MB-consistent way.[46,47]

The "physical asset accounting" of the SEEA, on the other hand, is designed to establish a framework for internationally comparable statistics on *stocks and flows in both physical and monetary terms*. The SEEA uses *economic accounting principles* that originate in the System of National Accounts (SNA), which measures economic activity and uses financial flows and monetary balances.[23] Alongside growing global environmental concerns, the SEEA has been gaining momentum, as the recently published "U.S. National Strategy to Develop Statistics for Environmental-Economic Decisions" illustrates.[48] The SEEA framework continues to evolve, and many details remain to be clarified: The section on "accounting for soil resources", for instance, mentions that further research on "spatially enabled data sets" is needed, as soil accounting can have many dimensions including soil type, composition (e.g. carbon and nutrient content), area (relevant for land use management), and volume (biological system functions).[48] This shows that our Critical Review and discussion of mineral resource accounting approaches is timely, and can inform an in-depth discussion on how to consistently account for geological stock changes.

Three key points need to be addressed to clarify (and potentially resolve) methodological differences and bridge the gap between the SEEA and MB-consistent geological stock accounting:

First, concerning the general *scope* for physical asset accounting, the SEEA notes that *"the asset boundary of the SEEA Central Framework is broader [than the SNA] and includes all natural resources and areas of land of an economic territory that may provide resources and space for use in*

economic activity." The "general principles" of the complementary SEEA Ecosystem Accounting (SEEA EA)[35] further specify: "*Ecosystem assets should be mutually exclusive, both conceptually and geographically. Thus, EAs should not overlap, either conceptually or geographically, and any area on the land or the sea floor, or any horizontal depth layer in the ocean, should be occupied by one and only one ecosystem asset."*

Second, concerning *MB-consistency*, the SEEA notes that *"in theory, mass and energy flows must balance"* (a physical accounting constraint), but simultaneously states that "physical asset accounts" can change in response to "*additions or reductions in the estimated available stock of a specific deposit or to changes in the categorization [...] based on changes in geologic information, technology, resource price or a combination of these factors*".[23]

And third, concerning *data resolution,* the SEEA notes *that "the coverage of individual assets does not extend to the individual elements that are embodied in the various natural and biological resources".*[23]

Given our definition of MB-consistent geological stock accounting, we here show for the three forementioned points that the current data reporting and SEEA asset accounting workflows, which use economic accounting methods and concepts, do not satisfy the 'physical accounting' requirements of MFA:

As for the first point (scope), the SEEA asset account balancing may not cover the entire physical space (i.e., "*all natural resources and areas of land of an economic territory that may provide resources and space for use in economic activity"*) and whatever is being balanced is thus not "collectively exhaustive" (cf. MECE-concept). This is illustrated by "discoveries of new stock"[23], which implicitly expand the spatial 3D system boundary that compounded the initial stock volume to include the new additional stock volume(s). Because the SEEA is not spatially explicit, it is unclear for the outside observer (and data user) that, and how exactly, the spatial system boundary changed. Moreover, it is inherently difficult to guarantee that reported resource and reserve numbers are "mutually exclusive" (MECE); while definitions across government and industry vary,[31,32,49] it is common that mineral "resources" numbers include "reserves",[31,32,49,50] which makes MB-consistent accounting difficult.

For the second point (MB-consistency), we observe that the SEEA 'reserves stock' is not defined in terms of purely *intrinsic*[51] physical material properties, but rather is a function of *extrinsic* socioeconomic valuation. Examples are changes of reported reserves stocks in response to expected

market price developments, or "upward reappraisal due to improvements in extraction technology", which violate MB-consistency because the spatiotemporal system boundary is not well constrained.

And for the last point (data resolution), it may be noted that both resource and reserves typically only quantify the content of the commodity of primary economic interest (e.g., copper grade and tonnage). Since neither the entire geological stock volume under consideration (including e.g., overburden), nor the whole-rock mineral/material composition thereof are reported, disclosed resources and reserves are not collectively exhaustive with respect to the material content.

Altogether, we show that economic accounting approaches are poorly suited for monitoring the physical reality and changing spatial characteristics. Our definitions and the outlined conceptual principles for MB-consistent geological stock accounting can thus be understood as inputs to the further development of the SEEA, natural capital accounting,[52] and physical monitoring.

Notes

Any use of trade, firm, or product names is for descriptive purposes only and does not imply endorsement by the U.S. Government.

S5. References:

1. Snyder, H. Literature review as a research methodology: An overview and guidelines. *J. Bus. Res.* **2019,** *104*, 333-339.
2. Bramer, W. M. Reference checking for systematic reviews using Endnote. *J Med Libr Assoc* **2018,** *106*, (4), 542-546.
3. Caputo, A.; Kargina, M. A user-friendly method to merge Scopus and Web of Science data during bibliometric analysis. *J. Mark. Anal.* **2022,** *10*, (1), 82-88.
4. Aria, M.; Cuccurullo, C. bibliometrix: An R-tool for comprehensive science mapping analysis. *J. Informetr.* **2017,** *11*, (4), 959-975.
5. Szomszor, M.; Adams, J.; Fry, R.; Gebert, C.; Pendlebury, D. A.; Potter, R. W. K.; Rogers, G. Interpreting Bibliometric Data. *Frontiers in Research Metrics and Analytics* **2021,** *5*, 628703.
6. Waltman, L.; van Eck, N. J. Field normalization of scientometric indicators. *Springer handbook of science and technology indicators* **2019**, 281-300.
7. Bader, G. D.; Forrester, B.; Ehlers, L.; Velliky, E.; MacDonald, B. L.; Linstädter, J. The Forgotten Kingdom: New investigations in the prehistory of Eswatini. *JOGA* **2021**, § 1–12.
8. Agricola, G. *De Re Metallica Libri XII*. Ludwig Königs: Basel, Switzerland, 1556.
9. USGS. *Principles of a resource/reserve classification for minerals - A revision of the classification system published as U.S. Geological Survey Bulletin 1450-A* U.S. Geological Survey: Reston, VA, 1980. DOI: 10.3133/cir831.
10. Von Carlowitz, H. C. *Sylvicultura Oeconomica - Oder Haußwirthliche Nachricht und Naturmäßige Anweisung zur Wilden Baum-Zucht*. Bey Johann Friedrich Brauns sel. Erben: Leipzig, 1732; Vol. 1.
11. Meadows, D. H.; Meadows, D. L.; Randers, J.; Behrens III, W. W. *The Limits to Growth: A Report for the Club of Rome's Project on the Predicament of Mankind*. Universe Books: New York, 1972.
12. Andrews, G. C.; Shaw, P.; McPhee, J. *Canadian Professional Engineering and Geoscience: Practice and Ethics*. 6 ed.; Nelson: Toronto, 2019.
13. U.S. Office of War Information. *A new method for classifying ore reserves has been adopted by the Bureau of Mines and the Geological Survey*; U.S. Government Printing Office: Washington DC, 1943. DOI: ark:/13960/t8sb4c74q.
14. UNHCR. Human Rights and Transnational Corporations and other Business Enterprises. United Nations, 2011.
15. NAEN. *The Russian Code for public reporting of exploration results, reserves and resources of solid minerals (The NAEN Code)*; The National Association of Subsoil Expertise (NAEN): Moscow, 2011.
16. United Nations General Assembly. The future we want. United Nations, 2012.
17. CRIRSCO. *Standard definitions*; Committee for Mineral Reserves International Reporting Standards (CRIRSCO) and International Council on Mining & Metals (ICMM): London, 2012.
18. JORC. *Australasian Code for Reporting of Exploration Results, Mineral Resources and Ore Reserves. The JORC Code*; 2012.
19. PERC. *PERC Reporting Standard 2013*; The Pan-European Reserves and Resources Reporting Committee: Brussels, Belgium, 2013.
20. UNEP. Minamata Convention on Mercury. UNEP, 2013.
21. CRIRSCO. *International Reporting Template for the public reporting of exploration results, mineral resources and mineral reserves*; Committee for Mineral Reserves International Reporting Standards (CRIRSCO) and International Council on Mining & Metals (ICMM): London, 2013.
22. CIM. *CIM Definition Standards for Mineral Resources & Mineral Reserves*; Canadian Institute of Mining, Metallurgy and Petroleum (CIM): Montreal, Canada, 2014.
23. United Nations; European Commission; Food and Agricultural Organization of the United Nations; International Monetary Fund; Organization for Economic Co-operation and Development; World Bank. *System of Environmental-Economic Accounting 2012: Central Framework*; United Nations: Washington, 2014. DOI: 10.5089/9789211615630.069.
24. United Nations General Assembly. Transforming our world: the 2030 Agenda for Sustainable Development. United Nations, 2015.
25. Ali, S. H.; Giurco, D.; Arndt, N.; Nickless, E.; Brown, G.; Demetriades, A.; Durrheim, R.; Enriquez, M. A.; Kinnaird, J.; Littleboy, A.; Meinert, L. D.; Oberhänsli, R.; Salem, J.; Schodde, R.; Schneider, G.; Vidal, O.; Yakovleva, N. Mineral supply for sustainable development requires resource governance. *Nature* **2017,** *543*, (7645), 367.
26. PERC. *PERC Reporting Standard 2017*; The Pan-European Reserves and Resources Reporting Committee: Brussels, Belgium, 2017.
27. SME. *The SME Guide for Reporting Exploration Results, Mineral Resources, and Mineral Reserves (The SME Guide)*; Society for Mining, Metallurgy & Exploration (SME): Englewood, CO, 2017; pp 97.

28. Norwegian Petroleum Directorate. *The Norwegian Petroleum Directorate's resource classification system 2016*; NPD-07-16; Norwegian Petroleum Directorate (NPD): Stavanger, Norway, 2018.
29. UNECE. *Specifications for the application of the United Nations Framework Classification for Resources to Anthropogenic Resources*; Working Group on Anthropogenic Resources of the Expert Group on Resource Classification, United Nations Economic Commission for Europe (UNECE): Geneva, Switzerland, 2018.
30. UNEA. Mineral Resource Governance. United Nations Environment Programme, 2019.
31. CRIRSCO. *International Reporting Template for the public reporting of exploration results, mineral resources and mineral reserves*; International Council on Mining & Metals (ICMM): London, 2019.
32. UNECE. *United Nations Framework Classification for Resources Update 2019*; ECE/ENERGY/125; United Nations Economic Commission for Europe: Geneva, Switzerland, 2019.
33. SAC. *GB/T 17766-2020: Classifications for mineral resources and mineral reserves*; Standardization Administration of the People's Republic of China (SAC): Beijing, China, 2020.
34. ICMM; UNEP; PRI. *Global Industry Standard on Tailings Management (GISTM)*; Nairobi, Kenya, 2020.
35. United Nations; European Commission; Food and Agricultural Organization of the United Nations; International Monetary Fund; Organization for Economic Co-operation and Development; World Bank. *System of Environmental-Economic Accounting - Ecosystem Accounting (SEEA EA)*; United Nations: Washington, D.C., 2021.
36. PERC. *PERC Reporting Standard 2021*; The Pan-European Reserves and Resources Reporting Committee: Brussels, Belgium, 2021.
37. UNEA. Environmental aspects of minerals and metals management. United Nations Environment Programme, 2022.
38. UNECE. *Draft UNRMS: Principles and Requirements*; ECE/ENERGY/GE.3/2022/6; United Nations Economic Commission for Europe UNECE: Geneva, Switzerland, 2022.
39. European Commission. Directive 2022/2464/EC of the European Parliament and of the Council of 14 December 2022 on Corporate Sustainability Reporting. European Parliament and the Council of the European Union, 2022.
40. European Commission. Proposal for a regulation of the European Parliament and of the Council establishing a framework for ensuring a secure and sustainable supply of critical raw materials. European Parliament and the Council of the European Union, 2023.
41. IFRS. Use of IFRS Standards around the world 2018. 2018. https://www.ifrs.org/use-around-the-world/use-of-ifrs-standards-by-jurisdiction/#analysis-of-use-of-ifrs-accounting-standards-around-the-world. (accessed 18.04.2023).
42. Gray, S. J.; Hellman, N.; Ivanova, M. N. Extractive Industries Reporting: A Review of Accounting Challenges and the Research Literature. *Abacus* **2019,** *55*, (1), 42-91.
43. IFRS. *Extractive Activities - Reserve and resource reporting*; International Accounting Standards Board (IASB): 2020.
44. VALMIN. *Australasian Code for Public Reporting of Technical Assessments and Valuations of Mineral Assets*; Valmin Committee: Carlton, Australia, 2015.
45. Simoni, M. U.; Aslaksen Aasly, K.; Eilu, P.; Schjødt, F. *Mintell4EU Deliverable D4.1. Case study review with guidance and examples for applying the UNFC to European mineral resources*; Geological Survey of Norway (NGU): Trondheim, Norway, 2021.
46. OECD. *Measuring material flows and resource productivity - Volume I. The OECD guide*; Organisation for Economic, Co-operation and Development: Paris, 2008.
47. EUROSTAT. *Economy-wide Material Flow Accounts. Handbook 2018 edition*; Eurostat: Luxembourg, 2018. DOI: 10.2785/158567
48. Office of Science and Technology Policy. *National strategy to develop statistics for environmental-economic decisions: A U.S. System of Natural Capital Accounting and Associated Environmental-Economic Statistics*; The White House Office of Science and Technology Policy (OSTP): Washington, 2023.
49. USGS. *Mineral commodity summaries 2019 - Appendix C —Reserves and Resources*; U.S. Geological Survey: Reston, VA, 2023. DOI: 10.3133/mcs2023.
50. Arndt, N. T.; Fontboté, L.; Hedenquist, J. W.; Kesler, S. E.; Thompson, J. F. H.; Wood, D. G. Section 1. Metals and Minerals, Now and in The Future. *Geochem. Perspect.* **2017,** *6*, (1), 3-17.
51. Pauliuk, S.; Majeau-Bettez, G.; Müller, D. B.; Hertwich, E. G. Toward a Practical Ontology for Socioeconomic Metabolism. *J. Ind. Ecol.* **2016,** *20*, (6), 1260-1272.
52. Bateman, I. J.; Mace, G. M. The natural capital framework for sustainably efficient and equitable decision making. *Nat. Sustainability* **2020,** *3*, (10), 776-783.
